# Innovative Eco-Friendly Microwave-Assisted Rapid Biosynthesis of Ag/AgCl-NPs Coated with Algae Bloom Extract as Multi-Functional Biomaterials with Non-Toxic Effects on Normal Human Cells

**DOI:** 10.3390/nano13142141

**Published:** 2023-07-24

**Authors:** Jeeraporn Pekkoh, Khomsan Ruangrit, Thida Kaewkod, Yingmanee Tragoolpua, Supawitch Hoijang, Laongnuan Srisombat, Antira Wichapein, Wasu Pathom-aree, Yasuo Kato, Guangce Wang, Sirasit Srinuanpan

**Affiliations:** 1 Department of Biology, Faculty of Science, Chiang Mai University, Chiang Mai 50200, Thailand; 2 Multidisciplinary Research Institute, Chiang Mai University, Chiang Mai 50200, Thailand; 3 Environmental Science Research Center, Faculty of Science, Chiang Mai University, Chiang Mai 50200, Thailand; 4 Department of Chemistry, Faculty of Science, Chiang Mai University, Chiang Mai 50200, Thailand; 5 Center of Excellent in Microbial Diversity and Sustainable Utilization, Chiang Mai University, Chiang Mai 50200, Thailand; 6 Biotechnology Research Center and Department of Biotechnology, Toyama Prefectural University, Toyama 939-0398, Japan; 7 CAS and Shandong Province Key Laboratory of Experimental Marine Biology, Center for Ocean Mega-Science, Institute of Oceanology, Chinese Academy of Sciences, Qingdao 266071, China; 8 Laboratory for Marine Biology and Biotechnology, Pilot National Laboratory for Marine Science and Technology (Qingdao), Qingdao 266237, China

**Keywords:** anticancer, antioxidant, biosynthesis, macroalgae, microwave, silver nanoparticles

## Abstract

Harmful algal blooms impact human welfare and are a global concern. *Sargassum* spp., a type of algae or seaweed that can potentially bloom in certain regions of the sea around Thailand, exhibits a noteworthy electron capacity as the sole reducing and stabilizing agent, which suggests its potential for mediating nanoparticle composites. This study proposes an eco-friendly microwave-assisted biosynthesis (MAS) method to fabricate silver nanoparticles coated with *Sargassum* aqueous extract (Ag/AgCl-NPs-ME). Ag/AgCl-NPs-ME were successfully synthesized in 1 min using a 20 mM AgNO_3_ solution without additional hazardous chemicals. UV–visible spectroscopy confirmed their formation through a surface plasmon resonance band at 400–500 nm. XRD and FTIR analyses verified their crystalline nature and involvement of organic molecules. TEM and SEM characterization showed well-dispersed Ag/AgCl-NPs-ME with an average size of 36.43 nm. The EDS results confirmed the presence of metallic Ag^+^ and Cl^−^ ions. Ag/AgCl-NPs-ME exhibited significant antioxidant activity against free radicals (DPPH, ABTS, and FRAP), suggesting their effectiveness. They also inhibited enzymes (tyrosinase and ACE) linked to diseases, indicating therapeutic potential. Importantly, the Ag/AgCl-NPs-ME displayed remarkable cytotoxicity against cancer cells (A375, A549, and Caco-2) while remaining non-toxic to normal cells. DNA ladder and TUNEL assays confirmed the activation of apoptosis mechanisms in cancer cells after a 48 h treatment. These findings highlight the versatile applications of Ag/AgCl-NPs-ME in food, cosmetics, pharmaceuticals, and nutraceuticals.

## 1. Introduction

Harmful macroalgae blooms, such as *Sargassum* blooms, have significant consequences for coastal ecosystems, economies, and human activities [1]. These blooms present considerable challenges. For example, macroalgal blooms can lead to excessive nutrient enrichment and oxygen depletion in aquatic ecosystems, causing harm to marine life, including fish, shellfish, and other aquatic organisms [1,2]. The rapid proliferation of harmful algal species can outcompete and displace native species, leading to a decline in biodiversity and disrupting the natural balance of marine ecosystems [1]. Blooms can release toxins and other harmful substances, leading to degraded water quality, making water unsuitable for drinking, recreational activities, and aquatic life [1,2,3]. In addition, some harmful algal species produce toxins that can pose health risks to humans through the consumption of contaminated seafood or direct exposure to toxins in the water during recreational activities [1]. However, recent studies have provided valuable insight into their potential for transformation into innovative solutions [2,3]. *Sargassum*, in particular, holds promise as a valuable resource for bioenergy production, primarily due to its high biomass productivity and unique biochemical composition [4]. Exploiting this potential can contribute to renewable energy goals and reduce dependence on fossil fuels. Additionally, *Sargassum* serves as an abundant source of bioactive compounds with diverse applications in pharmaceuticals and nutraceuticals. These bioactive compounds encompass antioxidants, anti-inflammatory agents, and antimicrobial substances, which offer exciting prospects for the development of novel drugs, functional foods, and dietary supplements [5,6]. Therefore, harnessing the potential of these compounds derived from *Sargassum* opens up avenues for advancements in healthcare and nutrition. By addressing the challenges posed by harmful macroalgae blooms and capitalizing on the multifaceted benefits of *Sargassum*, we can not only mitigate the negative impacts on coastal environments but also unlock new opportunities for sustainable energy production, pharmaceutical innovation, and improved nutrition.

Despite numerous studies highlighting the pharmaceutical and nutraceutical potential of different *Sargassum* products [7], challenges persist due to their limited solubility and bioavailability at the target organ [8,9]. Moreover, conventional formulations based on *Sargassum* extracts often exhibit toxicity to other organs and tissues [9]. To overcome these limitations, scientists have turned to nanotechnology-based approaches, utilizing clean, non-toxic, and environmentally friendly methods to develop biogenic metallic or metallic oxide nanoparticles (NPs), such as silver nanoparticles (AgNPs) [10], gold nanoparticles (AuNPs) [11], copper nanoparticles (CuNPs) [12], iron oxide nanoparticles (Fe_2_O_3_ or Fe_3_O_4_) [13], zinc oxide nanoparticles (ZnO NPs) [14], titanium dioxide nanoparticles (TiO_2_ NPs) [15], magnesium oxide nanoparticles (MgO NPs) [16], nickel nanoparticles (NiNPs) [17], selenium nanoparticles (SeNPs) [18], and cerium oxide nanoparticles (CeO_2_ NPs) [19]. Among the various types of nanoparticles, the green synthesis of AgNPs using plant extracts is very well known [10] and AgNPs have emerged as highly promising materials for a wide range of biomedical applications. These applications include tissue regeneration, cosmetics, industries, healthcare, medical device coatings, and biosensors [20]. The unique properties of AgNPs, such as their small size, large surface area, and enhanced reactivity, make them ideal candidates for targeted drug delivery, diagnostic imaging, antimicrobial agents, and wound healing, among other applications. However, these properties of AgNPs depend on the method of their preparation and are not their general features [10]. By incorporating *Sargassum*-derived bioactive compounds into AgNPs, researchers can potentially overcome the solubility and bioavailability limitations of *Sargassum* products while simultaneously taking advantage of the multifunctional properties of AgNPs. This innovative approach holds great promise in enhancing the efficacy and safety of pharmaceutical and nutraceutical applications, addressing the existing challenges, and providing new possibilities for utilizing *Sargassum* in diverse biomedical fields.

Various synthetic methodologies are available for producing AgNPs, including chemical [21], photochemical [22], electrochemical [23], microemulsion [24], and microwave techniques [25]. However, many of these techniques involve the use of hazardous substances and require stringent reaction conditions, leading to chemical toxicity and environmental pollution [25]. To address these concerns and adhere to the principles of green chemistry, the biosynthesis of AgNPs has gained prominence due to its simplicity, eco-friendly nature, and cost-effectiveness. Furthermore, AgNPs synthesized through biological methods exhibit desirable properties, such as high-water solubility, biocompatibility, and reduced toxicity [26]. However, compared to chemical approaches, biological processes generally have slower reaction kinetics. This limitation can be overcome by incorporating microwave irradiation into the biosynthesis process, enabling rapid and efficient green synthesis of AgNPs. The use of microwave-assisted synthesis (MAS) offers several advantages, including faster reaction times, lower energy consumption, increased product yield, and uniform heat distribution within the reaction medium [27]. Numerous studies have demonstrated the successful production of AgNPs using microwave-assisted methods [27,28]. Specifically, several research groups have reported the biosynthesis of AgNPs using aqueous extracts of macroalgae *Sargassum* (AgNPs-ME), which act as both reducing and capping agents [29,30]. These AgNPs-ME have shown considerable potential for various biotechnological applications due to their antioxidant properties [11], enzyme inhibitory activity [31], and anticancer properties [32]. However, the synthesis of AgNPs-ME has predominantly been conducted using traditional biosynthesis methods, with limited research exploring the application of microwave-assisted synthesis (MAS) for the green synthesis of AgNPs-ME. Furthermore, comprehensive investigations regarding the characteristics of these nanoparticles and their biological potential evaluations are scarce in the existing literature. Therefore, further research in this area is warranted to explore the full potential of microwave-assisted synthesis for the green synthesis of AgNPs-ME and to gain a deeper understanding of their properties and potential applications in biotechnology.

The objective of this study was to develop a microwave-assisted biosynthesis method for the production of AgNPs using a *Sargassum* macroalgae extract (Ag/AgCl-NPs-ME) as the sole reducing and stabilizing agent, eliminating the need for additional chemical agents. The research focused on investigating the impact of the reaction conditions, specifically the synthesis times and AgNO_3_ concentration, on the synthesis process of Ag/AgCl-NPs-ME. Multiple analytical techniques were employed to thoroughly characterize the synthesized Ag/AgCl-NPs-ME, including UV–vis spectroscopy, scanning electron microscopy (SEM), transmission electron microscopy (TEM), Fourier-transform infrared spectroscopy (FTIR), and X-ray diffraction (XRD). In addition to characterization, the antioxidant properties of the synthesized Ag/AgCl-NPs-ME were evaluated to determine their potential as antioxidants. The enzyme inhibition potential of Ag/AgCl-NPs-ME against tyrosinase and angiotensin-converting enzyme (ACE) was investigated to measure their potential health benefits in vitro. Furthermore, this study makes a significant contribution by meticulously assessing the in vitro cytotoxicity of the synthesized Ag/AgCl-NPs-ME against normal human cells, A375 skin cancer cells, A549 lung cancer cells, and Caco-2 colon cancer cells.

## 2. Materials and Methods

### 2.1. Preparation of Macroalgal Extract

The dried biomass of *Sargassum* spp., obtained from the Applied Algal Research Laboratory at Chiang Mai University, Thailand, underwent a week-long process of solar drying until a consistent weight was achieved. Subsequently, the dried biomass was finely ground into powder form and subjected to the extraction process using a modified version of the water extraction technique described by Balarama et al. [29]. Specifically, 20 g of the powdered biomass was mixed with 200 mL of distilled water and heated to 60 °C for 20 min. The resulting extract was then subjected to centrifugation at 6000 rpm for 20 min, followed by filtration through Whatman filter paper No. 1. Finally, the extract was stored at a temperature of 4 °C to ensure its preservation for future use. The extract contained polysaccharides at 730.54 mg/g extract, proteins at 135.90 mg/g extract, and total phenolic at 1.25 mg GAE/g extract.

### 2.2. Biosynthesis of Silver Nanoparticles

Silver nanoparticles (Ag/AgCl-NPs-macroalgal extract; Ag/AgCl-NPs-ME) were synthesized using two different methods: a rapid microwave-assisted technique and a conventional continuous stirring approach. In the microwave-assisted synthesis, a solution containing 18 mL of 1 mM silver nitrate (AgNO_3_) and 2 mL of macroalgal extract underwent microwave irradiation (Sharp R-221F-K) at 800 W power, 2450 MHz frequency, and 100 °C temperature for various synthesis durations ranging from 0 to 4 min. The formation of Ag/AgCl-NPs-ME was monitored by analyzing the reaction mixture at specific time intervals (0, 1, 2, 3, and 4 min) within the 300–600 nm range using a UV–vis spectrophotometer. Additionally, the impact of different AgNO_3_ concentrations (ranging from 1 to 25 mM) was investigated under the optimized synthesis time. In the conventional method, Ag/AgCl-NPs-ME were produced by combining 18 mL of the 1 mM AgNO_3_ solution with 2 mL of macroalgal extract and continuously stirring the mixture in darkness for 24 h at room temperature. Subsequently, a larger-scale synthesis of Ag/AgCl-NPs-ME was performed based on the optimized synthesis time and AgNO_3_ concentration. The resulting Ag/AgCl-NPs-ME pellet was collected and washed by centrifugation at 6000 rpm for 40 min. To obtain a powdered form, the pellet was then lyophilized and stored in a desiccator at room temperature for further characterization studies and evaluation of its biological potential.

### 2.3. Characterization of Synthesized Ag/AgCl-NPs-ME

The formation of Ag/AgCl-NPs-ME was assessed using the Agilent Cary 60 UV–vis spectrophotometer, operating within a wavelength range of 300–600 nm. An X-ray diffraction (XRD) analysis was conducted using the Rigaku SmartLab instrument, which was equipped with Cu Ka radiation (λ = 1.5406 Å). The scanning range for 2θ spanned from 10° to 90° with scan steps of 0.01°, enabling the identification of the nanoparticle phases. The morphology of the sample was examined using both transmission electron microscopy (TEM) with a JEOL JEM-2010 instrument and scanning electron microscopy (SEM) with a JEOL JSM-IT800 instrument. TEM also facilitated the capture of the selected area electron diffraction (SAED) pattern of the nanoparticles. The chemical composition of the nanoparticles was determined through energy dispersive X-ray spectroscopy (EDS) analysis, which was performed in conjunction with the SEM. Additionally, the functionality of the sample was evaluated using attenuated total reflectance-Fourier transform infrared spectroscopy (ATR-FTIR) with a Bruker TENSOR27 instrument, covering the scanning wavenumber range of 400 cm^−1^ to 4000 cm^−1^.

### 2.4. Biological Potentials

#### 2.4.1. 2,2-Diphenyl-1-Picrylhydrazyl (DPPH) Radical Scavenging Activity

The ability to scavenge DPPH radicals was assessed using a modified protocol based on Ruangrit et al.’s method [6]. A 1.5 mL microcentrifuge tube was utilized to combine 200 µL of Ag/AgCl-NPs-ME (5–40 µg/mL) with 400 µL of a 0.3 mM methanolic solution of DPPH. The mixture was then incubated at room temperature, shielded from light, while continuously agitated at 300 rpm. After a 20-min incubation period, the absorbance of the solution was measured at 517 nm. The DPPH radical scavenging activity was determined using the following equation:

DPPH radical scavenging activity (%) = {[A − (B − C)]/A} × 100 (1)

where A, B, and C correspond to the absorbance values of the control blank (lacking Ag/AgCl-NPs-ME), the Ag/AgCl-NPs-ME solution, and the Ag/AgCl-NPs-ME solution without DPPH, respectively. The half maximal inhibitory concentration (IC_50_) value was calculated as the concentration of Ag/AgCl-NPs-ME required to inhibit the radical scavenging activity by 50%.

#### 2.4.2. 2,2′-Azino-Bis(3-Ethylbenzothiazoline-6-Sulfonic Acid) (ABTS) Radical Scavenging Activity

The ABTS radical scavenging activity was evaluated using a modified method based on Pekkoh et al.’s approach [33]. The preparation of the ABTS working reagent involved combining 1 mL of 0.015 mM ABTS with 1 mL of 2.45 mM potassium persulfate (K_2_S_2_O_8_) in a light-protected environment for 16 h at room temperature. Subsequently, the resulting mixture was diluted with distilled water to achieve an ABTS working solution with an absorbance (OD734) of 0.700 ± 0.020. In a 96-well plate, 200 µL of the ABTS solution and 10 µL of the Ag/AgCl-NPs-ME solution (10–50 µg/mL) were carefully added, thoroughly mixed, and shielded from light for 10 min at 37 °C. Afterward, the absorbance was measured at 734 nm. The determination of the ABTS radical scavenging activity was calculated using Equation (2):

ABTS radical scavenging activity (%) = {[A − (B − C)]/A} × 100 (2)

where A represents the absorbance of the control blank (lacking Ag/AgCl-NPs-ME), B denotes the absorbance of the Ag/AgCl-NPs-ME solution, and C signifies the absorbance of the Ag/AgCl-NPs-ME solution without ABTS. The concentration at which the tested Ag/AgCl-NPs-ME inhibited 50% of the radical scavenging activity was determined as the IC_50_.

#### 2.4.3. Ferric-Reducing Antioxidant Power Activity

The capacity of Ag/AgCl-NPs-ME to convert Fe^3+^-TPTZ to Fe^2+^-TPTZ was assessed using the ferric-reducing antioxidant power (FRAP) test, adapted from a technique developed by Pekkoh et al. [34]. To prepare the FRAP reagent, a mixture was prepared by combining 100 mL of a 300 mM acetic acid buffer (pH 3.6), 10 mL of a 40 mM hydrochloric acid (HCl) solution containing 10 mM 2,4,6-tripyridyl-s-triazine (TPTZ), and 10 mL of a 20 mM iron(III) chloride hexahydrate (FeCl_3_6H_2_O) solution. Following a 30-min incubation of Ag/AgCl-NPs-ME (10–50 µg/mL) in a dark chamber at 37 °C, 150 µL of the FRAP reagent was added to each well of a 96-well plate. The absorbance was then measured at 593 nm. A standard curve of ferrous sulfate heptahydrate (FeSO_4_·7H_2_O) was created by adding the FRAP reagent to a range of Fe^2+^ solutions of known concentrations (0.36–2.88 mM FeSO^4^·7H_2_O in 155 µL working reaction volume = 21–177 µg Fe^2+^/mL), which allows the enhancement of the Fe^2+^ concentration by the Ag/AgCl-NPs-ME to be calculated, thereby determining “FRAP activity (µg Fe^2+^/mL)”. The IC_50_ was determined as the concentration of the Ag/AgCl-NPs-ME that resulted in a 50% reduction of the Fe^3+^-TPTZ.

#### 2.4.4. Tyrosinase Inhibitory Activity

The inhibitory effect of Ag/AgCl-NPs-ME on the tyrosinase activity was evaluated using the spectrophotometric method outlined by Pekkoh et al. [34]. The test was conducted in a 96-well microplate by combining 264 µL of 0.85 mM l-3,4-dihydroxyphenylalanine (L-DOPA) substrate in 20 mM phosphate buffer (pH 6.8) with 50 µL of Ag/AgCl-NPs-ME (5–25 µg/mL). The mixture was allowed to incubate at room temperature for 10 min. Subsequently, 6 µL of the tyrosinase enzyme derived from mushrooms (1000 U/mL) was added to initiate the reaction. The absorbance at 492 nm was then measured. The degree of tyrosinase inhibition was determined using Equation (3).

Tyrosinase inhibition activity (%) = {[A − (B − C)]/A} × 100 (3)

where A denotes the absorbance of the control blank (without Ag/AgCl-NPs-ME), B represents the absorbance of the Ag/AgCl-NPs-ME solution, and C signifies the absorbance of the Ag/AgCl-NPs-ME solution in the absence of the tyrosinase enzyme. To quantify the concentration at which Ag/AgCl-NPs-ME inhibited 50% of the tyrosinase activity, the IC_50_ was determined.

The experiment was repeated by varying the concentrations of the L-DOPA substrate (0.50, 1.00, 1.25, and 1.50 mM) while maintaining the Ag/AgCl-NPs-ME concentration at 20 and 25 µg/mL. A Lineweaver–Burk plot analysis (also known as a double reciprocal plot) was used to examine how Ag/AgCl-NPs-ME inhibits the tyrosinase enzyme. Using the Michaelis–Menten kinetic equation (Equation (4)), we were able to determine both the Michaelis constant (K_m_) and the maximal response rate (V_max_):

V_0_ = V_max_ [S]/K_m_ + [S] (4)

where V_0_ represents the initial velocity of the reaction, V_max_ corresponds to the maximum reaction rate, K_m_ signifies the Michaelis constant, and [S] denotes the concentration of the substrate.

#### 2.4.5. Angiotensin-Converting Enzyme (ACE) Inhibitory Activity

The ACE-inhibitory activity was assessed by modifying the method described by Phinyo et al. [35]. In a 96-well microplate, 31 µL of 50 mM sodium borate buffer (pH 8.3) with 0.3 M NaCl (SBBS) was added to each well. Subsequently, 5 µL of angiotensin-I converting enzyme (ACE) derived from rabbit lungs (200 mU/mL) was added. The control reaction consisted of 10 µL of either Ag/AgCl-NPs-ME (0.25–5.00 g/mL) or SBBS. The reaction was initiated by adding a final volume of 59 µL of 5 mM hippuryl-L-histidyl-L-leucine (HHL) substrate. Two additional wells were prepared: one without ACE and the inhibitor Ag/AgCl-NPs-ME (Bi), and another without ACE and HHL (Bs). After an initial incubation period of 1 h at 37 °C, 100 µL of 200 mM sodium tetraborate, 50 µL of 10 mM sodium sulfite, and 50 µL of 3.4 mM TNBS (2,4,6-trinitrobenzenesulfonic acid) were added to each well. Following another 20 min of incubation at 37 °C, the absorbance of the mixtures was measured at a wavelength of 420 nm. The extent of the ACE inhibition was determined using Equation (5):

ACE inhibition activity (%) = {[(C − Bi) − (S − Bs)]/(C − Bi)} × 100(5)

where the absorbance values are represented as follows: C for the control (100% activity), S for the sample (Ag/AgCl-NPs-ME inhibitor), Bi for the blank inhibitor (HHL alone), and Bs for the blank sample (Ag/AgCl-NPs-ME alone). The IC_50_ value was calculated as the concentration of Ag/AgCl-NPs-ME required to inhibit ACE activity by 50%.

HHL substrate doses of 1, 2, 3, and 4 mM and Ag/AgCl-NPs-ME concentrations of 0.25 and 2.5 µg/mL were used in the subsequent experiments. By analyzing a Lineweaver–Burke plot, we were able to determine the mechanism by which Ag/AgCl-NPs-ME inhibited the ACE enzyme. As discussed in Section 2.4.4, the K_m_ and V_max_ were determined using the Michaelis–Menten kinetic equation (Equation (4)).

#### 2.4.6. Anticancer Activity

##### Cytotoxicity of Cancer Cells and Normal Cells

The cytotoxicity of Ag/AgCl-NPs-ME was assessed using the 3-(4,5-dimethylthiazolyl-2)-2,5-diphenyltetrazolium bromide (MTT) test [35]. Human cancer cell lines, including A375 melanoma cells, A549 lung adenocarcinoma cells, Caco-2 colorectal carcinoma cells, and Vero normal cells, were pre-cultured in a 5% CO_2_ atmosphere at 37 °C for 24 h. The pre-cultivation medium for the A549 cells, Caco-2 cells, and Vero cells consisted of Dulbecco’s modified eagle medium (DMEM) supplemented with 10% heat-inactivated fetal bovine serum (FBS), penicillin (100 Units/mL), and streptomycin (100 µg/mL). The A375 cells were maintained in a medium containing DMEM with pyruvate, 1% 4-(2-hydroxyethyl)-1-piperazineethanesulfonic acid (HEPES), 10% FBS, 100 U/mL penicillin, and 100 µg/mL streptomycin. The cells were then seeded at a density of 10^5^ cells/mL in 96-well plates and incubated at 37 °C in a 5% CO_2_ atmosphere for 24 h. After filling the wells with Ag/AgCl-NPs-ME at various concentrations, the plates were incubated for 48 h at 37 °C in a 5% CO_2_ atmosphere. Following a 4 h incubation period, a solution of 3-(4,5-dimethylthiazol-2-yl)-2,5-diphenyltetrazolium bromide (MTT) at a concentration of 2 mg/mL was added to 30 µL of the solution. After incubation, the blue formazan was dissolved by adding 200 µL of dimethyl sulfoxide (DMSO) to each well and thoroughly mixing the contents. The absorbance was measured at two different wavelengths, 540 nm and 630 nm. The percentage of live cells was calculated using Equation (6):

Cell viability (%) = (A_treated cells_/A_control_) × 100 (6)

where A_control_ represents the absorbance of the culture cells serving as the control and A_treated cells_ represents the absorbance of the culture cells that received treatment. The concentration of the Ag/AgCl-NPs-ME that inhibited fifty percent of the culture cells was determined as the IC_50_.

##### Deoxyribonucleic Acid (DNA) Fragmentation Analysis using DNA Ladder Assay

The DNA ladder assay [35] was employed to investigate the DNA fragmentation in the cells following treatment with Ag/AgCl-NPs-ME. Specifically, cancer cells were incubated at a concentration of 2 × 10^5^ cells/mL in 24-well plates at 37 °C with 5% CO_2_ for 24 h. After 48 h of incubation with Ag/AgCl-NPs-ME at 37 °C and 5% CO_2_, the cells were harvested by pelleting and then trypsinized using 0.05% trypsin-ethylenediaminetetraacetic acid (trypsin-EDTA). The resulting cell pellets were then lysed with 30 µL of a lysis solution consisting of 10 mM tris (hydroxymethyl) aminomethane hydrochloride (Tris-HCl), 2.5 mM ethylenediamine tetraacetic acid (EDTA), 100 mM sodium chloride (NaCl), and 1% sodium dodecyl sulfate (SDS) at pH 8.0. This lysing procedure was repeated three times to ensure complete cell lysis. The lysate was thoroughly mixed using a vortex mixer, followed by the addition of 5 M NaCl, proteinase K, and ribonuclease A (RNase A) at concentrations of 10 mg/mL each. The combined solution was then incubated for three hours at 37 °C. Subsequently, the DNA fragmentation analysis was performed on a 2% agarose gel at 60 volts for a duration of 3 h. The resulting DNA fragments were visualized using a UV transilluminator.

##### DNA Fragmentation Analysis Using TUNEL Assay

DNA fragmentation induced by apoptosis can be detected using the well-established TUNEL (terminal deoxynucleotidyl transferase dUTP nick end labeling) test [35], employing the DNA fragmentation imaging kit from Merck, Germany, which incorporates terminal deoxynucleotidyl transferase and fluorescein-labeled dUTP. In this study, cancer cells were treated with Ag/AgCl-NPs-ME for 48 h at a concentration of 2 × 10^5^ cells/mL. After collecting the cells, they were subjected to three washes with phosphate-buffered saline (PBS, pH 7.4). Following the washes, the cells were fixed with 4% paraformaldehyde (100 µL) and incubated at room temperature for 10 min. Subsequently, the fixed cells were treated with 0.1% Triton X-100 (100 µL) and incubated at room temperature for 20 min. The cells were then washed twice with phosphate-buffered saline (pH 7.4). After centrifugation, the cells were treated with an enzyme solution containing terminal deoxynucleotidyl transferase (TdT) (45 µL) and incubated at 37 °C in a 5% CO_2_ atmosphere for 1 h. Following the incubation, the cells were treated with a nuclei dye combination (Hoechst 33,342) (150 µL) and incubated in the dark at room temperature for 15 min. The excess reagent was removed by centrifugation at 5000× *g* and 4 °C for 5 min. Fluorescent DNA fragments were visualized using an inverted fluorescence microscope (ECLIPSE Ts2R-FL, Nikon, Tokyo, Japan). Finally, the resulting pellets were resuspended using ProLong^TM^ gold antifade mountant from Life Technologies, Camarillo, CA, USA.

## 3. Results and Discussion

### 3.1. Microwave-Assisted Rapid Biosynthesis of Silver Nanoparticles

Conventional methodologies for the production of biogenic silver nanoparticles (AgNPs) typically involve prolonged shaking in the absence of light, resulting in a significant time investment. However, microwave-assisted synthesis (MAS) offers several advantages over traditional techniques. Notably, MAS enables shorter synthesis durations, reduced energy consumption, and improved efficiency in achieving uniform dispersion of nanoparticles. As a result, MAS has gained widespread adoption for the synthesis of AgNPs using various reducing agents and stabilizing ligands, as highlighted in studies by Revathi et al. [27] and Acar et al. [28]. Building upon this knowledge, our research aimed to utilize the MAS approach to fabricate AgNPs coated with organic molecules derived from macroalga *Sargassum* biomass. To achieve this objective, we systematically optimized the key process parameters, such as the synthesis time and AgNO_3_ concentration, to ensure the desired outcome was attained with precision and efficacy.

The synthesis of Ag/AgCl-NPs using macroalgae extract-mediated MAS methods encompasses various potential mechanisms. While the specific mechanism may vary based on the extract and experimental conditions employed, the following provides a general overview of the process: (I) Reduction of Ag^+^ ions: macroalgae *Sargassum* extracts contain diverse organic compounds with inherent reducing properties. These compounds, including polysaccharides, proteins, polyphenols, flavonoids, and other organic molecules, interact with Ag^+^ ions present in the reaction solution. Through redox reactions, these compounds facilitate the conversion of Ag^+^ ions to AgNPs [29]. (II) Stabilization and capping: the organic compounds in the extract assume a critical role in stabilizing and capping the formed AgNPs. They adsorb onto the nanoparticle surface, preventing aggregation and ensuring colloidal stability. These capping agents function as a protective layer, impeding nanoparticle growth and agglomeration [36]. (III) Formation of AgCl: in the presence of chloride ions (Cl^−^), either naturally occurring or provided by the macroalgae *Sargassum* extract, the reaction can yield AgCl as a byproduct. Cl^−^ ions react with Ag^+^ ions, leading to the precipitation of AgCl on the surface of AgNPs. This process contributes to the development of Ag/AgCl-NPs, where AgCl serves as a constituent of the nanoparticle structure [36,37]. (IV) Microwave-assisted heating: the use of microwave irradiation in the synthesis process can generate localized heating, leading to rapid and efficient reactions. Microwave energy can accelerate the reduction of silver ions and promote the nucleation and growth of Ag/AgCl-NPs [27,28]. It is important to acknowledge that the specific mechanism and contributions of individual components within the extract can vary depending on factors such as the experimental conditions, concentrations, and intricate interactions [38].

**Figure 1:**
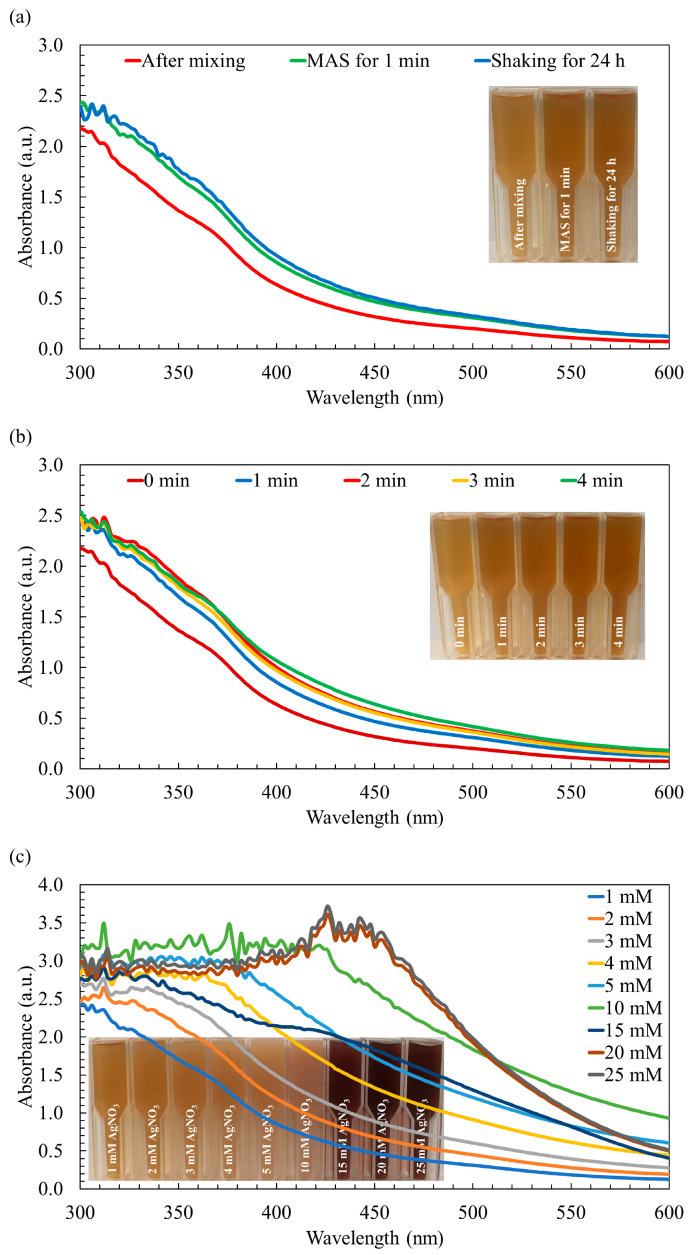
The UV–visible absorption spectra of the synthesized Ag/AgCl-NPs-ME. (**a**) The effect of microwave-assisted synthesis compared to the conventional method employing continuous stirring, (**b**) the effect of different synthesis times, and (**c**) the effect of varying AgNO_3_ molarity.

Upon the combination of the AgNO_3_ solution with the macroalga *Sargassum* extract, a remarkable transformation occurred, initiating the formation of AgNPs and leading to a visible change in color. Initially, the mixture of the *Sargassum* aqueous extract and AgNO_3_ solution displayed a light brown hue. Subsequently, employing both synthesis methods, the color of the mixture transitioned from light brown to medium brown, as visually depicted in Figure 1a. This perceivable shift in color, recognized as a characteristic optical phenomenon associated with noble metals, indicates the occurrence of surface plasmon resonance, thereby serving as an indicative feature of AgNPs synthesis [28]. In line with the observations presented in Figure 1a, the color and UV–visible absorption spectra of the AgNPs synthesized through the rapid MAS for 1 min demonstrated similar characteristics to those synthesized using the conventional approach involving continuous stirring over a 24-h period. Typically, AgNPs exhibit a characteristic peak within the wavelength range of 400 nm to 500 nm [37]. However, in the context of this study, the synthesized AgNPs did not manifest the broader peak typically observed within this range. This phenomenon can be attributed to the incomplete reduction of the Ag^+^ ions, indicating that the synthesis of the AgNPs was not fully accomplished.

Although the UV–visible absorption spectra of the AgNPs synthesized using both methods exhibit similarity, the utilization of MAS leads to a notable reduction in the duration of the synthesis process and a decrease in energy consumption. By subjecting the reaction mixture to microwave irradiation, the polysaccharides present can undergo degradation, leading to the release of reducing sugars. These reducing sugars play a crucial role in efficiently reducing AgNO_3_ to AgNPs, facilitating their formation [39]. Moreover, the by-products resulting from the degradation of the reducing agents act as capping agents, contributing to the enhanced stability of the nanoparticles. Furthermore, the rapid and localized heating generated by the microwave irradiation at the reaction sites significantly accelerates the reaction rate [25]. However, no noticeable changes in absorbance and color were observed after irradiation periods of 2, 3, and 4 min compared to the 1 min irradiation period, as shown in Figure 1b. This observation suggests that the availability of the Ag^+^ ions may have reached a limit, and/or the complete conversion of Ag^+^ to Ag was achieved [25]. Considering the substantial significance of shorter synthesis durations and reduced energy consumption, particularly in the context of large-scale production of AgNPs, the irradiation time was methodically established and maintained at a fixed duration of 1 min for the purposes of this study. 

It is important to note that when increasing the concentration of AgNO_3_ from 1 mM to 15 mM, a clear and noticeable change in the color was observed, transitioning from a medium brown shade to a darker brown hue, as depicted in Figure 1c. It is also worth noting that at a AgNO_3_ concentration of 20 mM, a distinctive peak attributed to the surface plasmon oscillations of AgNPs appeared within the wavelength range of 420–450 nm, coinciding with the deepest brown color observed in the reaction mixture. The intensity of this AgNP peak demonstrated a significant enhancement as the AgNO_3_ concentration increased, reaching its maximum intensity at 20 mM. This can be attributed to the successful completion of the synthesis process, resulting in the formation of AgNPs. However, when the concentration was further increased to 25 mM AgNO_3_, the peak intensity became comparable to that of 20 mM AgNO_3_ (Figure 1c). This observation suggests that the quantity of macroalga *Sargassum* extract used might have been insufficient to fully cap all the Ag^+^ ions present in the mixture, which aligns with findings reported by Moshahary and Mishra [40]. Consequently, 20 mM AgNO_3_ was selected as the optimal concentration for subsequent characterization studies and the evaluation of its biological potential.

The differences observed in the spectra of 15 mM AgNO_3_ compared to 10 mM and 20 mM AgNPs could be attributed to various factors related to nanoparticle synthesis and behaviors. For example, different concentrations of the AgNO_3_ used might lead to variations in the size distribution of the nanoparticles. Smaller nanoparticles may have different optical properties and absorbance spectra compared to larger ones [41]. Higher concentrations could influence the shape of the nanoparticles formed. AgNPs with varying shapes (e.g., spherical, rod-shaped) may exhibit different spectral profiles [42]. In addition, the kinetics of nanoparticle formation and aggregation might differ at various concentrations, affecting the overall spectral features [43].

Previous studies have indicated the presence of polysaccharides, proteins, flavonoids, polyphenols, and phenolic compounds in *Sargassum* aqueous extract, suggesting their potential involvement in the bioreduction of Ag^+^ ions to AgNPs and their subsequent capping within the solution [36,44]. Although the exact mechanism of green synthesis for AgNPs remains unclear, it is hypothesized that the organic molecules of *Sargassum* play a crucial role in the formation and stabilization of AgNPs. These compounds are likely responsible for binding Ag^+^ ions, facilitating their reduction and nucleation to form suitable nuclei. The reduction of Ag^+^ ions to Ag° occurs through a one-step, one-electron oxidation-reduction mechanism, with the aqueous fraction of this macroalgae acting as a green reducing agent [29].

### 3.2. Characterization of AgNPs Coated with Macroalgal Extract

**Figure 2:**
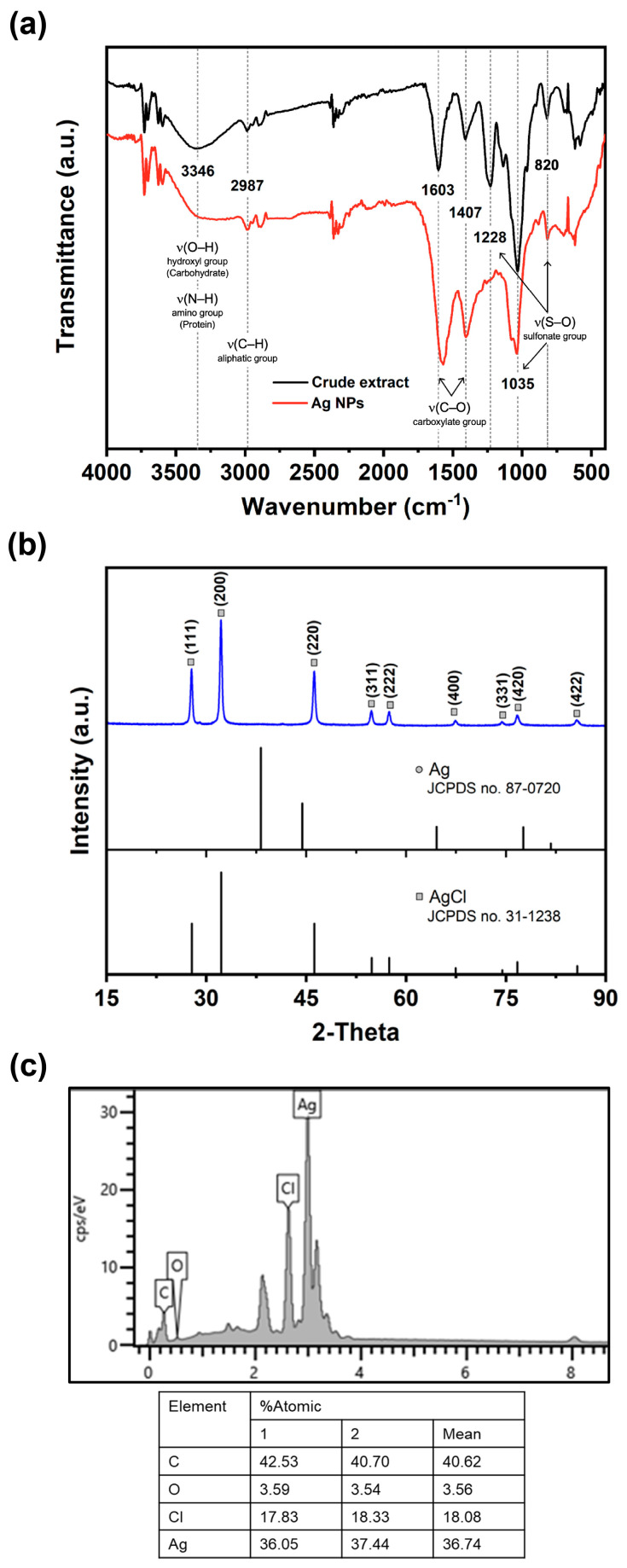
FTIR spectrum (**a**), XRD patterns (**b**), and EDX spectrum (**c**) of synthesized Ag/AgCl-NPs-ME.

Fourier transform infrared (FTIR) spectroscopy was employed to investigate the interaction between various functional groups involved in the formation of AgNPs. The resulting absorption spectrum exhibited distinct characteristic peaks at different wavenumbers: 820 cm^−1^, 1035 cm^−1^, 1228 cm^−1^, 1407 cm^−1^, 1603 cm^−1^, 2987 cm^−1^, and 3403 cm^−1^ (Figure 2a). The peaks observed at 820 cm^−1^, 1035 cm^−1^, and 1228 cm^−1^ are attributed to the presence of sulfonate groups (S–O) in sulfate polysaccharides [45]. Additionally, the absorption peaks at 3346 cm^−1^, corresponding to amino groups (N–H) present in the proteins and peptides, were evident in the absorption spectrum of the AgNPs [36]. The absorption peaks at 3346 cm^−1^ and 1603 cm^−1^ indicate the stretching vibrations of the O–H and C–O groups, respectively, associated with the carbohydrate group in the AgNPs [46]. Furthermore, the presence of C–H stretching vibrations at 2987 cm^−1^, characteristic of an aliphatic group in the aromatic components, was also detected [36]. The presence of these distinct FTIR absorption peaks provides confirmation that the synthesized AgNPs were coated with organic molecules derived from *Sargassum*, which likely encompassed carbohydrates, proteins, and phenolic compounds. Identical peaks have been recorded for the FTIR spectrum of AgNPs coated with the *Sargassum* extract [29,30].

The crystal quality of the AgNPs was assessed through X-ray diffraction (XRD) spectrum analysis, as depicted in Figure 2b. The resulting chromatic spectrum exhibited distinct and well-defined Bragg’s reflection peaks at specific 2θ degree values: 27.79°, 32.20°, 46.23°, 54.80°, 54.47°, 67.39°, 74.41°, 76.67°, and 85.65°. These peaks corresponded to the crystallographic planes of (111), (200), (220), (311), (222), (400), (331), (420), and (422), respectively. The identification of these planes provided further confirmation of the face-centered cubic structure of both Ag and AgCl, aligning with the standards established by the Joint Committee on Powder Diffraction Standards (JCPDS) database, specifically file numbers 87–0720 and 31–1238. These results indicate that the MAS technique not only resulted in the formation of AgNPs, but also facilitated the simultaneous generation of AgCl-NPs. It is plausible that the presence of Cl^−^ in the marine *Sargassum* extract facilitated their interaction with AgNO_3_, leading to the formation of AgCl. Over time, the AgCl species underwent a reduction, giving rise to the formation of AgCl-NPs, while other Ag^+^ ions contributed to the formation of Ag/AgCl-NPs. Similar observations were observed in Ag/AgCl-NPs synthesized by algae [37,47]. Aside from the XRD analysis, verification of the AgNPs’ formation was also conducted through energy-dispersive X-ray spectroscopy (EDX) analysis. The EDS spectrum of AgNPs is presented in Figure 2c. The spectra exhibited prominent peaks corresponding to Ag atoms and Cl atoms, thus confirming the successful synthesis of Ag/AgCl-NPs nanohybrids. Moreover, the observed atomic ratio of Ag to the Cl element was found to exceed 1:1 (Figure 2c), potentially indicating the co-existence of metallic AgNPs with AgCl-NPs. As a result, the AgNPs produced in this study were designated as Ag/AgCl-NPs-macroalgal extract (Ag/AgCl-NPs-ME).

**Figure 3:**
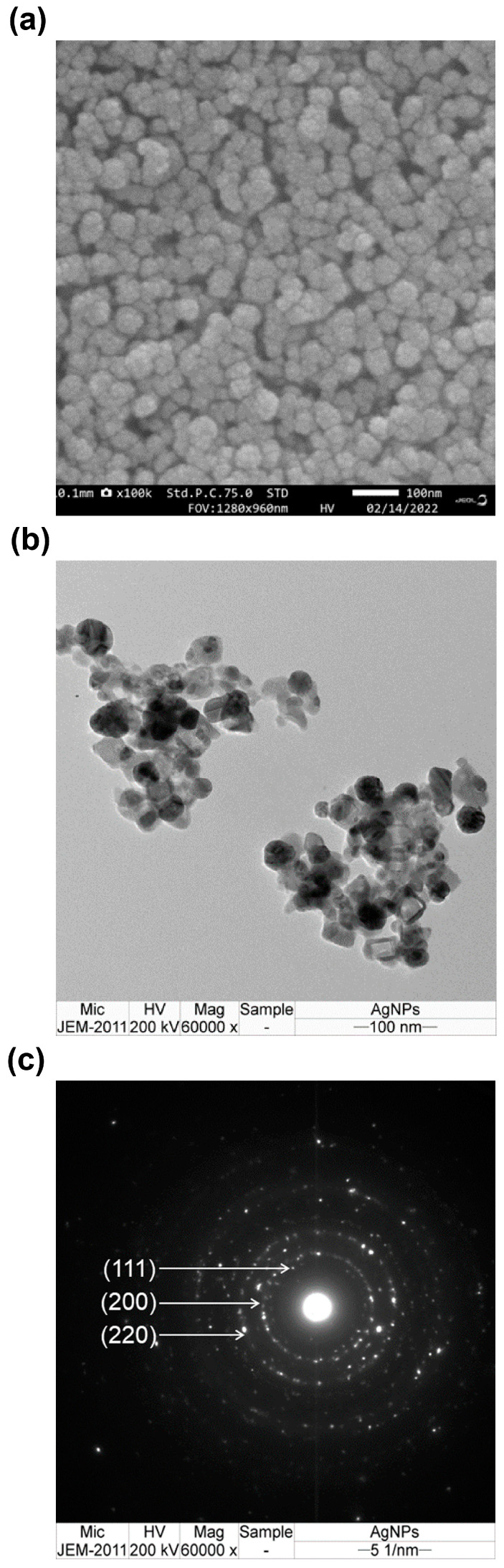
SEM image (**a**), TEM image (**b**), and SAED image (**c**) of synthesized Ag/AgCl-NPs-ME.

The external morphology of the synthesized Ag/AgCl-NPs-ME was examined using the scanning electron microscopy (SEM) technique. The SEM images revealed a predominant spherical shape for the synthesized Ag/AgCl-NPs-ME. The particles exhibited a uniform distribution, with some agglomerates displaying a closely compacted arrangement, and their size was determined to be below 100 nm (Figure 3a). The observed agglomeration is likely attributable to the presence of hydrogen bonding, which was also confirmed by the analysis of the FTIR spectra [38]. In order to gain deeper insight into the morphology and size distribution of the Ag/AgCl-NPs-ME, transmission electron microscopy (TEM) was employed. The TEM analysis provided a more detailed view of the topography and size distribution of the Ag/AgCl-NPs-ME, revealing a well-defined crystalline structure. The samples consisted of a mixture of spherical nanoparticles (small ~10 nm and large ~172 nm) with varying degrees of aggregation (Figure 3b). Moreover, the crystallographic nature of the Ag/AgCl-NPs-ME was confirmed through the analysis of the selected area electron diffraction (SAED) pattern, which exhibited distinct circular rings corresponding to the Bragg’s diffraction of the (111), (200), and (220) planes (Figure 3c). The TEM observations of the Ag/AgCl-NPs-ME are consistent with the findings reported in several previous studies [36,38].

**Figure 4:**
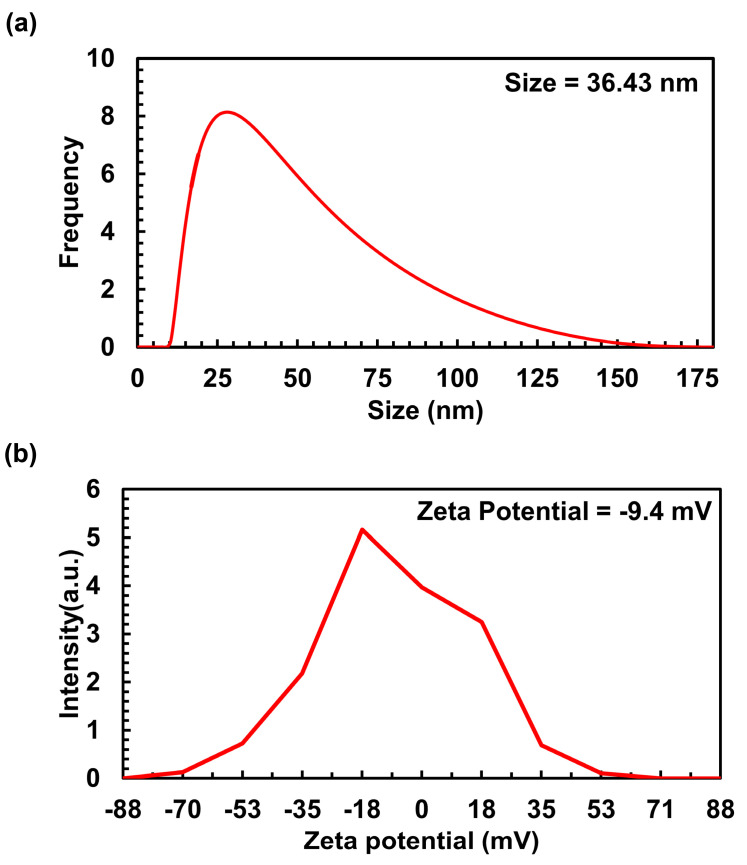
DLS (**a**) and zeta potential (**b**) of synthesized Ag/AgCl-NPs-ME.

The size and distribution characteristics of Ag/AgCl-NPs-ME were assessed using dynamic light scattering (DLS) analysis. The DLS analysis clearly revealed a particle size range of 10 to 175 nm, with an average size of 36.43 nm for Ag/AgCl-NPs-ME (Figure 4a). The findings from these measurements match those from the TEM study, thus establishing consistency in the findings. The zeta potential (ZP) is a parameter that elucidates the motion of nanoparticles in an electric field, taking into account their charge and location. It serves as an indicator of the electrostatic forces between neighboring and similar particles in a colloidal solution. In this study, the ZP value of Ag/AgCl-NPs-ME was determined to be −9.4 mV (Figure 4b), signifying enhanced dispersion and heightened stability of the nanoparticles. This characteristic makes them promising materials for potential biomedical applications [38].

### 3.3. Biological Potentials

#### 3.3.1. DPPH Radical Scavenging Activity

**Figure 5:**
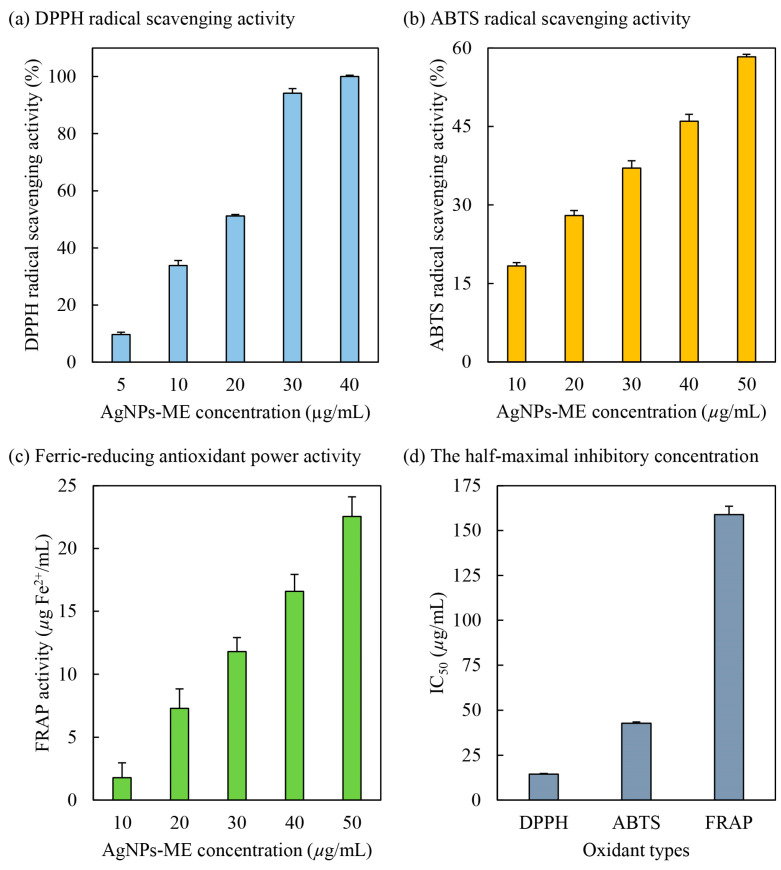
Antioxidant activities, including DPPH radical scavenging activity (**a**), ABTS radical scavenging activity (**b**), and ferric-reducing antioxidant power (FRAP) activity (**c**), and the half-maximal inhibition concentration (IC_50_; µg/mL) (**d**) of Ag/AgCl-NPs-ME.

DPPH (2,2-diphenyl-1-picrylhydrazyl) is a well-known free radical that is capable of inducing oxidative stress in human cells, contributing to the development of various diseases [20]. In this study, the potential of Ag/AgCl-NPs-ME (silver/silver chloride nanoparticles synthesized using macroalgal extracts) as a scavenger of DPPH radicals was evaluated. The biosynthesized Ag/AgCl-NPs-ME was utilized, with DPPH serving as the source of free radicals. The results, depicted in Figure 5, demonstrate a positive correlation between the scavenging activity against DPPH radicals and the concentration of Ag/AgCl-NPs-ME. Specifically, at concentrations of 5, 10, 20, 30, and 40 µg/mL, Ag/AgCl-NPs-ME exhibited scavenging potentials of 9.68%, 33.83%, 51.17%, 94.16%, and 100%, respectively. These findings highlight the concentration-dependent DPPH radical scavenging ability of Ag/AgCl-NPs-ME. Moreover, Ag/AgCl-NPs-ME exhibited remarkable antioxidant properties, as indicated by its IC_50_ value of 14.49 µg/mL (Figure 5). It is noteworthy that AgNO_3_ did not show any observable DPPH radical scavenging activity (data not shown). These results suggest that Ag/AgCl-NPs-ME likely possess potent proton donors, such as hydroxyl groups found in *Sargassum* metabolites (e.g., proteins, polysaccharides, and phytochemicals). These proton donors can effectively interact with unstable free radicals of DPPH, converting them into more stable products and inhibiting the initiation step in oxidative chain reactions [6]. Consequently, Ag/AgCl-NPs-ME shows promise as a DPPH inhibitor, as it is capable of mitigating oxidative stress by scavenging DPPH free radicals. Similar findings have been reported by Singh et al. [20], Kiran et al. [48], and Vijayakumar et al. [49], who demonstrated the effective neutralization of DPPH free radicals by AgNPs coated with various extracts, such as black cumin (*Nigella sativa*) seed extracts, *Cassia fistula* pod extracts, and *Eucalyptus tereticornis* leaf extracts, yielding IC_50_ values ranging from 10 to 63 µg/mL. However, it should be noted that the antioxidant capacity of AgNPs relies on the properties of the organic molecules bound or capped on their surfaces [48].

#### 3.3.2. ABTS Radical Scavenging Activity

ABTS (2,2′-azinobis-(3-ethylbenzothiazoline-6-sulfonic acid)) is known to induce oxidative stress in human cells [20]. It is widely employed to evaluate the antioxidant properties of compounds that possess hydrogen-donating and chain-breaking capabilities [50] and operates through an electron transfer mechanism [51]. The ABTS radical scavenging activity of Ag/AgCl-NPs-ME was assessed, and the results are presented in Figure 5. With increasing concentrations of Ag/AgCl-NPs-ME from 10 to 50 µg/mL, the ABTS radical scavenging activity exhibited a corresponding increase from 18.35% to 58.30%, indicating a progressive dose-dependent protective effect of Ag/AgCl-NPs-ME. Similar findings have been reported by Elemike et al. [52], Otunola and Afolayan [53], and Tanase et al. [54], who observed dose-dependent ABTS radical scavenging activity of AgNPs coated with various extracts, such as *Costus afer* leaf extract and *Picea abies* L. stem bark extract and spice blend, wherein an increase in the concentration of AgNPs correlated with enhanced ABTS free radical scavenging activity. Consistent with the results in Figure 5, Ag/AgCl-NPs-ME exhibited antioxidant potential by effectively scavenging ABTS, as evidenced by an IC_50_ value of 42.74 µg/mL. These findings align with previous studies conducted by Badmus et al. [10], Ajayi and Afolayan [50], and Chokshi et al. [55], who reported IC_50_ values ranging from 14 to 124 µg/mL for AgNPs coated with different extracts, including *Annona muricata* leaf extract, alkalinized *Cymbopogon citratus* Stapf extract, and de-oiled microalgal water extract. This suggests that the stability of ABTS free radicals is achieved through the acceptance of hydrogen ions from organic molecules present on AgNPs. Therefore, Ag/AgCl-NPs-ME not only scavenges DPPH free radicals, but also stabilizes ABTS free radicals, demonstrating its potential as an inhibitor of oxidative stress for cellular protection.

#### 3.3.3. Ferric-Reducing Antioxidant Power (FRAP) Activity

The ferric-reducing antioxidant power (FRAP) assay is a commonly employed method to assess the ability of antioxidant substances to reduce the Fe^3+^-TPTZ complex to the Fe^2+^-TPTZ complex, facilitated by electron-donating antioxidants [34]. This process involves the donation of a hydrogen atom, leading to the breakdown of the free radical chain [56]. As shown in Figure 5, Ag/AgCl-NPs-ME demonstrates notable electron-donating antioxidant or reductant properties by effectively reducing Fe^3+^ to Fe^2+^, resulting in FRAP activities of 1.78, 7.30, 11.78, 16.59, and 22.55 µg Fe^2+^/mL for concentrations of 10, 20, 30, 40, and 50 µg Ag/AgCl-NPs-ME/mL, respectively. These findings establish a direct relationship between the concentration of Ag/AgCl-NPs-ME and its FRAP activity. Similar observations have been reported for AgNPs coated with extracts derived from *Nothapodytes nimmoniana* (Graham) Mabb. fruits [51], *Cassia roxburghii* leaves [57], and *Cordia myxa* [58]. These studies indicate that the reducing potential increases proportionally with the quantity of AgNPs, indicating dose-dependent antioxidant activity of Ag/AgCl-NPs-ME. The effectiveness of Ag/AgCl-NPs-ME as an antioxidant can be attributed to the presence of organic molecules encapsulated on the surface of the nanoparticles. Furthermore, the IC_50_ value of Ag/AgCl-NPs-ME was determined to be 158.87 µg/mL (Figure 5), aligning with previous reports on AgNPs synthesized using extracts from *Nothapodytes nimmoniana* (Graham) Mabb. fruits [51], *Cassia roxburghii* leaves [57], *Cordia myxa* [58], and *Caesalpinia sappan* aqueous extract [59].

#### 3.3.4. Tyrosinase Inhibition Activity and Kinetic Study

**Figure 6:**
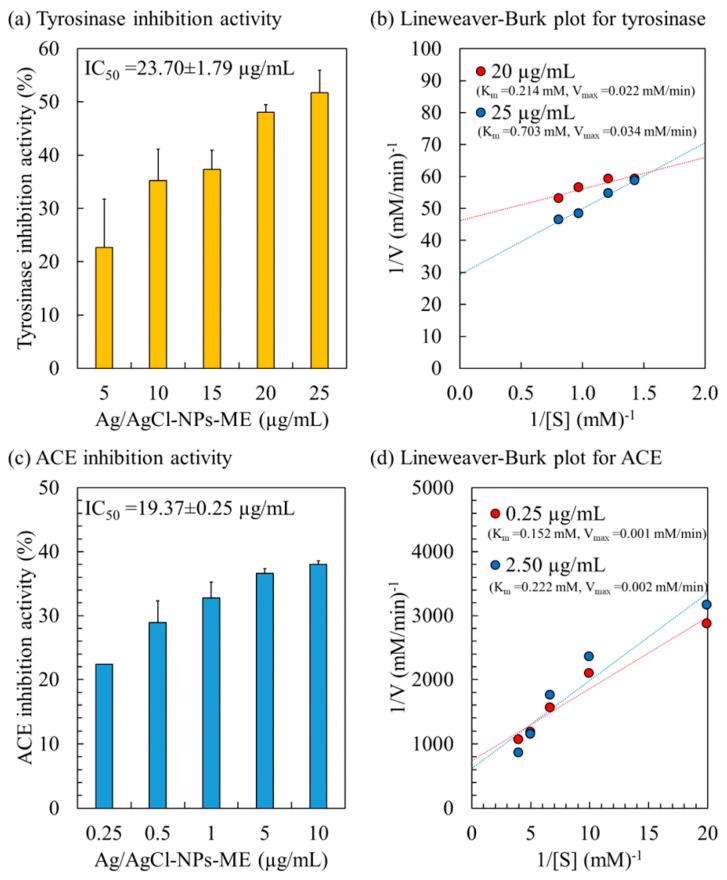
Enzyme inhibitory activity and kinetic study of Ag/AgCl-NPs-ME on tyrosinase enzyme (**a**,**b**) and ACE enzyme (**c**,**d**).

The tyrosinase enzyme, a copper-containing catalyst, plays a vital role in the synthesis of melanin in human skin cells, utilizing L-DOPA as its substrate. Overexpression of tyrosinase leads to an excessive production of melanin, resulting in various skin issues, such as dark spots, premature aging, age spots, freckles, and dryness [34]. The inhibition of the tyrosinase enzyme is a critical parameter for evaluating skin-lightening activity [60]. Figure 6 presents the tyrosinase inhibition activity of Ag/AgCl-NPs-ME. Within the concentration range of 5 to 25 µg/mL, the tyrosinase inhibition activity ranges from 22.64% to 51.72%, demonstrating a dose-dependent effect with an IC_50_ value of 23.70 µg/mL. These findings align with previously reported IC_50_ values for AgNPs synthesized using the aqueous extract of *Hovenia dulcis* fruits [61], *Bidens frondosa* [62], and *Sideritis* aqueous extract [63], which range from 15 to 83 µg/mL. The variation in IC_50_ values may be attributed to the utilization of different organic molecules in the synthesis of AgNPs.

To gain a better understanding of the kinetics and mechanism of tyrosinase inhibition, the inhibition pattern of Ag/AgCl-NPs-ME on the tyrosinase enzyme was assessed using a Lineweaver–Burke plot (or double reciprocal plot), as illustrated in Figure 6. The Ag/AgCl-NPs-ME demonstrated a mixed inhibition pattern on the tyrosinase enzyme, as evidenced by an increase in both K_m_ and V_max_ with escalating concentrations of Ag/AgCl-NPs-ME. This indicates that Ag/AgCl-NPs-ME can bind to the tyrosinase enzyme regardless of whether the enzyme has already bound the L-DOPA substrate. This suggests that Ag/AgCl-NPs-ME binds to a distinct site on the enzyme, separate from the L-DOPA binding site, resulting in the formation of complexes such as [Tyrosinase]–[L-DOPA]–[Ag/AgCl-NPs-ME] and [Tyrosinase]–[Ag/AgCl-NPs-ME]. Such inhibition often arises from allosteric action, wherein the inhibitor attaches to a specific location on the enzyme, causing conformational changes that reduce the affinity for substrate binding [34]. Consequently, Ag/AgCl-NPs-ME holds promising potential as a tyrosinase inhibitor for application in cosmetic and skincare products.

#### 3.3.5. ACE Inhibitory Activity and Kinetic Study

The angiotensin-converting enzyme (ACE) plays a pivotal role in actively regulating blood pressure through distinct mechanisms within the renin–angiotensin–aldosterone system (RAAS) and the kinin nitric oxide system (KNOS) [33]. ACE activity significantly contributes to the development of hypertension, a notable risk factor associated with cardiovascular disease [35]. Consequently, inhibiting ACE has emerged as a recognized therapeutic approach for hypertension [33,35]. The inhibitory activity of Ag/AgCl-NPs-ME on ACE demonstrated a dose-dependent trend, with an IC_50_ value of 19.37 µg/mL (Figure 6). Analysis of the inhibition pattern revealed a mixed type of inhibition for Ag/AgCl-NPs-ME characterized by an increase in both K_m_ and V_max_ (Figure 6). This suggests that AgNPs-ME can interact with ACE at a distinct site away from the substrate (HHL), resulting in the formation of complexes such as [ACE enzyme]–[HHL]–[Ag/AgCl-NPs-ME] and [ACE enzyme]–[Ag/AgCl-NPs-ME], thereby functioning as an ACE inhibitor. These findings support the potential of Ag/AgCl-NPs-ME as a viable alternative for antihypertensive therapy.

In a related study, Talapko et al. [64] reported that AgNPs not only exhibit effective ACE inhibition, but also induce endothelial vasodilation, leading to enhanced blood flow to the heart, which holds therapeutic implications for hypertension [65]. Nonetheless, it is crucial to acknowledge the potential side effects associated with AgNPs-ME, as highlighted by Ramirez-Lee et al. [66]. The toxicity of AgNPs primarily arises from the partial solubilization and release of Ag ions [67]. Animal studies have demonstrated that the side effects of AgNPs depend on various factors, including size, dosage, and duration of exposure, aligning with the considerations for all pharmaceutical agents and medical devices [65]. Recently, Gomes et al. [68] proposed surface modification of AgNPs through conjugation with polymeric materials, such as polyethylene glycol (PEG)-poly lactide (PLA), chitosan, silica-based compounds, and poly(lactic-co-glycolic acid) (PLGA), as a strategy to mitigate the toxicity associated with AgNPs. Further investigations encompassing surface modification, toxicity testing, and clinical evaluation of Ag/AgCl-NPs-ME are required to ensure their efficacy and safety as therapeutic interventions.

#### 3.3.6. Anticancer Activity

**Figure 7:**
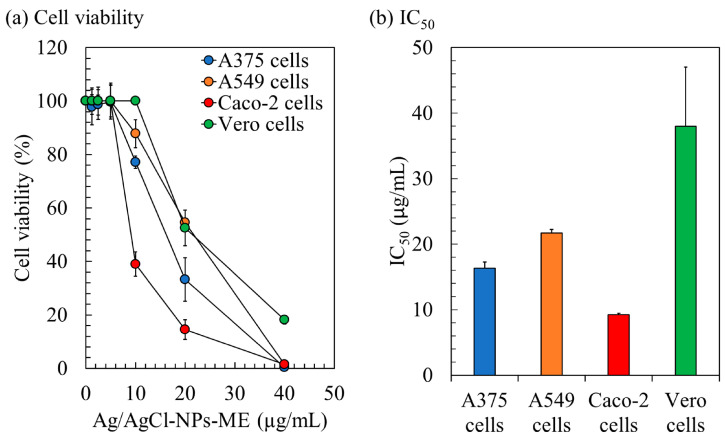
The impact of the Ag/AgCl-NPs-ME on the cytotoxicity of skin cancer A375 cells, lung cancer A549 cells, and colon cancer Caco-2 cells. The treatment was compared with control cells, represented by normal Vero cells, over a period of 48 h. Cell viability was assessed using the 3-(4,5-Dimethylthiazol-2-yl)-2,5-diphenyltetrazolium bromide reduction (MTT) assay, as shown in (**a**), and the results were calculated to enable comparisons with the control cells, as depicted in (**b**).

The cytotoxicity of Ag/AgCl-NPs-ME was evaluated using the MTT assay, which involves the reduction of 3-(4,5-Dimethylthiazol-2-yl)-2,5-diphenyltetrazolium bromide. The assessment focused on three human cancer cell lines (A375, A549, and Caco-2) and normal Vero cells for comparison. The results reveal a consistent decline in the cell viability as the concentration of Ag/AgCl-NPs-ME increases (Figure 7). After a 48 h treatment period, Ag/AgCl-NPs-ME exhibited notable antiproliferative activity, as demonstrated by IC_50_ values of 16.33 µg/mL (A375), 21.70 µg/mL (A549), 9.25 µg/mL (Caco-2), and 38.00 µg/mL (Vero) (Figure 7). The low IC_50_ values signify that Ag/AgCl-NPs-ME exerts potent anticancer effects at relatively low concentrations, resulting in a significant inhibition of proliferation across all three cancer cell lines, while exerting a minimal impact on normal cells. It is essential to acknowledge that there are no established standard IC_50_ values or ranges for comparative analysis. Typically, AgNPs are expected to exhibit lower IC_50_ values against cancer cells than normal cells. In a similar vein, Gomes et al. [68] suggested that AgNPs, which selectively target cancer cells without posing harm to normal cells, hold promise as a model for the development of cancer treatment strategies.

Previous studies have employed the selectivity index (SI) as a means to evaluate the preferential targeting of compounds between cancer and normal cells [35]. The SI was determined by dividing the average IC_50_ value in the normal cell line by the IC_50_ value in the cancer cell line obtained from each independent experiment. For Ag/AgCl-NPs-ME, the SI values ranged from 1.78 to 4.11, indicating its greater efficacy against cancer cells while demonstrating reduced toxicity towards normal cells. Notably, an SI value ≥ 10 is commonly regarded as indicative of a promising candidate that merits further investigation [69]. Potential anticancer samples may be categorized with a lower SI value of 3, as suggested by Weerapreeyakul et al. [70]. An SI value of 2 is indicative of a potentially useful alternative or supplemental therapy for cancer [71]. Furthermore, Krzywik et al. [72] suggested that compounds with an SI greater than 1.0 exhibit a preferential effectiveness against cancer cells compared to normal cells. These findings highlight the potential utility of Ag/AgCl-NPs-ME as an inhibitor for cancer treatment. Notably, the heightened sensitivity of cancer cells observed in this study compared to normal cells holds promise for the development of effective anticancer applications with reduced systemic toxicity.

In the literature, AgNPs have demonstrated promising potential in exerting effects on cancer cells through a range of mechanisms [68]. The precise mechanisms of action may vary depending on factors such as the nanoparticles’ size, shape, surface properties, and concentration, and the characteristics of the targeted cancer cells [73]. The following elucidates several methods by which AgNPs might influence cancer cells: (I) Cytotoxicity: AgNPs possess cytotoxic effects on cancer cells, potentially inducing cell death by activating apoptosis, a regulated process of cellular demise. Consequently, these nanoparticles impede uncontrolled cell proliferation within cancerous tissues [74]. (II) Anti-proliferative activity: AgNPs have been observed to impede cell proliferation in cancer cells. By interfering with cellular processes governing cell division and growth, these nanoparticles can impede or halt the proliferation of cancer cells [75]. (III) DNA damage: AgNPs have the capability to inflict DNA damage in cancer cells. This DNA damage disrupts critical cellular functions and contributes to the inhibition of cancer cell growth and proliferation [42]. (IV) Generation of reactive oxygen species (ROS): AgNPs have the capacity to generate reactive oxygen species (ROS) within cancer cells. Elevated ROS levels induce oxidative stress, thereby instigating cytotoxic effects and promoting apoptosis in cancer cells [76]. (V) Disruption of cell signaling pathways: AgNPs may disrupt essential signaling pathways vital for the survival and growth of cancer cells [77]. By interfering with these pathways, the nanoparticles impede cancer cell proliferation and survival.

**Figure 8:**
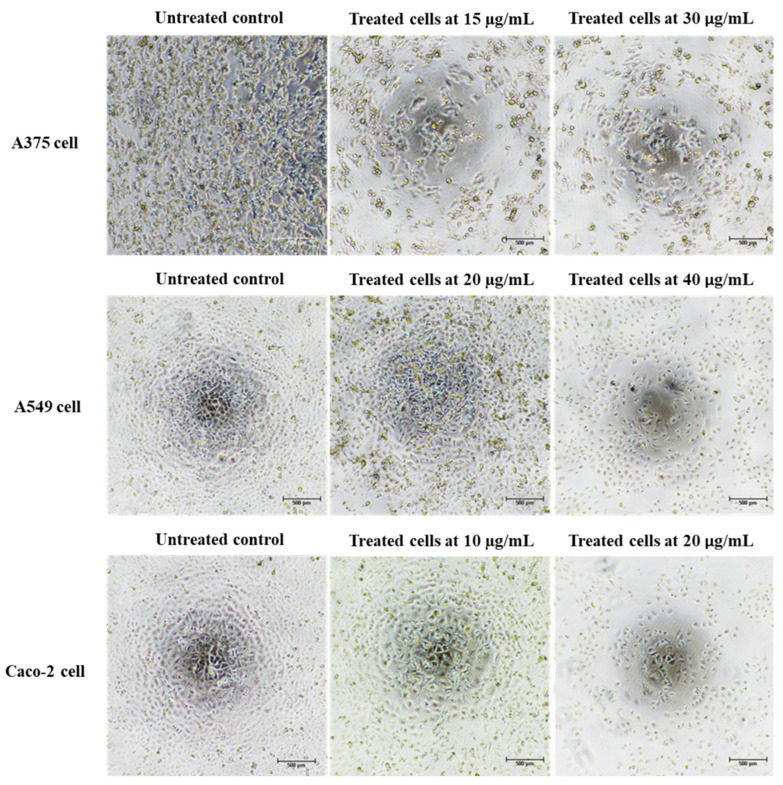
The morphological changes observed in skin cancer A375 cells, lung cancer A549 cells, colon cancer Caco-2 cells, and normal Vero cells following a 48 h treatment with the Ag/AgCl-NPs-ME. A comparison was made between the treated cells and the control cells (which remained untreated) to assess the impact of the extract on cellular morphology.

**Figure 9:**
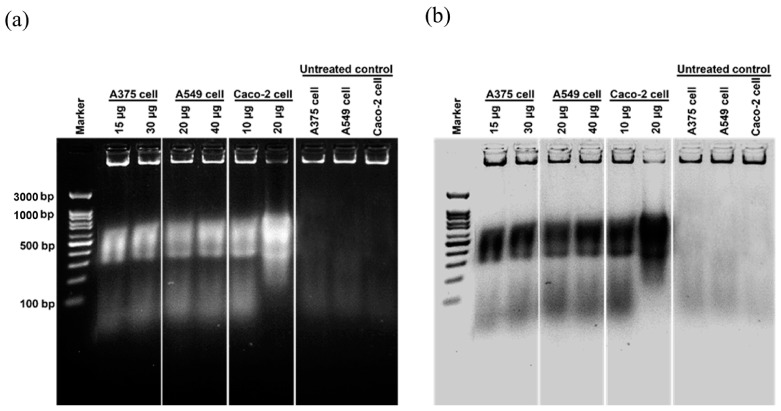
The analysis of deoxyribonucleic acid (DNA) fragmentation in skin cancer A375 cells, lung cancer A549 cells, and colon cancer Caco-2 cells following a 48 h treatment with the Ag/AgCl-NPs-ME. The treated cells are compared to the control cells, represented by normal Vero cells, using agarose gel electrophoresis, as shown in (**a**). Additionally, the agarose gel is inverted to display the DNA fragmentation pattern in black and white, as depicted in (**b**).

Cancer is characterized by uncontrolled cellular proliferation and the suppression of apoptosis in the affected cells. Traditional approaches to cancer treatment have predominantly focused on inhibiting cell division and inducing apoptosis to eliminate malignant cells [35]. In this investigation, we conducted a thorough examination of the morphological alterations associated with apoptosis to elucidate the apoptotic effects exerted by Ag/AgCl-NPs-ME. Following a 48 h exposure to varying concentrations of Ag/AgCl-NPs-ME, the treated cancer cells exhibited diminished cellular adhesion, cellular shrinkage, and a rounded morphology in comparison to the untreated cells (Figure 8). The concentration-dependent response of Ag/AgCl-NPs-ME further validated the presence of apoptosis-related features, including cellular shrinkage, membrane blebbing, nuclear chromatin condensation, the formation of apoptotic bodies, and engulfment by neighboring cells (Figure 8). These findings are consistent with previous studies conducted by Phinyo et al. [35] and Pekkoh et al. [78]. Additionally, we investigated the activation of DNA fragmentation, a distinctive hallmark of apoptotic cell death, to corroborate the occurrence of apoptosis. Through agarose gel electrophoresis analysis (Figure 9), we detected DNA fragments ranging from 200 to 1000 bp in cancer cells treated with Ag/AgCl-NPs-ME. Notably, as the concentration of Ag/AgCl-NPs-ME increased, more pronounced DNA bands were observed, indicating the ability of this nanomaterial to induce apoptosis-associated DNA fragmentation across all three types of cancer cells.

**Figure 10:**
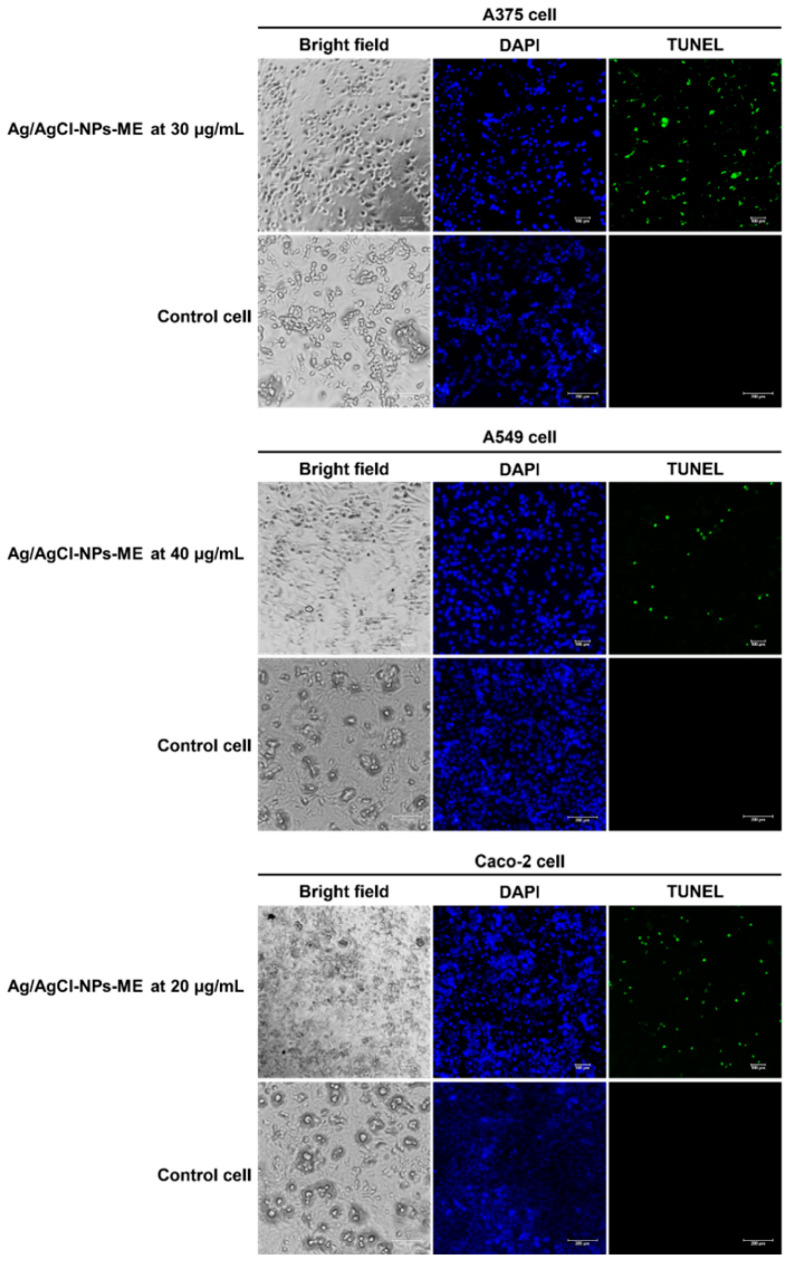
The results of the terminal deoxynucleotidyl transferase dUTP nick end labeling (TUNEL) assay conducted on skin cancer A375 cells, lung cancer A549 cells, and colon cancer Caco-2 cells following a 48 h treatment with the Ag/AgCl-NPs-ME. The treated cells are compared to control cells, represented by normal Vero cells. The cells were stained with 4′,6-diamidino-2-phenylindole (DAPI) and TUNEL and observed using a fluorescent microscope.

The terminal deoxynucleotidyl transferase dUTP nick end labeling (TUNEL) assay is a method for confirming DNA fragmentation in apoptotic processes [34]. In this study, we examined the impact of Ag/AgCl-NPs-ME on DNA damage in three distinct types of cancer cells. To achieve this, we employed 4′,6-diamidino-2-phenylindole (DAPI) and TUNEL fluorescent dyes to label the cancer cells following a 48 h treatment with Ag/AgCl-NPs-ME. DAPI staining, which emits blue fluorescence, facilitated the visualization of the cell viability by highlighting the cellular nuclei. Conversely, TUNEL labeling allowed for the identification of DNA degradation. In this process, the labeled dUTP was integrated into the free hydroxyl termini resulting from genomic DNA fragmentation, catalyzed by terminal deoxynucleotidyl transferase (TdT) [34]. As illustrated in Figure 10, the results demonstrate green fluorescence in the nuclei of all three cancer cell types subsequent to treatment with Ag/AgCl-NPs-ME, indicating positive TUNEL labeling. In contrast, the nuclei of the untreated cancer cells did not exhibit such fluorescence, suggesting that all three cancer cell types experienced similar levels of DNA fragmentation, which was further corroborated by agarose gel electrophoresis analysis. These findings highlight the potential of Ag/AgCl-NPs-ME for prospective cancer treatment applications. However, additional investigations are needed to analyze the expression of apoptosis-related genes, thereby enhancing our comprehension of the molecular mechanisms governing the apoptotic pathways in cancer cells subjected to Ag/AgCl-NPs-ME treatment. Such understanding will enable a comprehensive evaluation of their potential for clinical applications in cancer treatment.

After conducting a comprehensive investigation, it should be highlighted that the Ag/AgCl-NPs-ME exhibits promising potential as a prospective solution for scavenging harmful free radicals, mitigating enzyme-related diseases, and acting as a therapeutic agent for cancer. However, it is imperative to acknowledge that this study was confined to in vitro experimentation. To gain deeper insight into the response of the Ag/AgCl-NPs-ME within an organism, whether it be a laboratory animal or a human, further research incorporating in vivo assays is required. In vivo studies enable the examination of the actual impact on living organisms, whereas clinical trials or medical studies can be conducted using either in vitro or in vivo approaches. These methodologies share a common objective of advancing our understanding of illness, disease, and the normal biological functioning of the human body. Thus, it is crucial to emphasize the necessity for both in vitro and in vivo investigations to ascertain the potential outcomes when employing the Ag/AgCl-NPs-ME in practical applications.

## 4. Conclusions

*Sargassum* spp., a macroalgal species known to cause harmful blooms in certain regions of the sea around Thailand, contains organic compounds (i.e., polysaccharides, proteins, flavonoids, polyphenols, and phenolic compounds) with functional groups (i.e., hydroxyl (−OH), carbonyl (C=O), amino (−NH_2_), carboxyl (−COOH), and phenolic (−C_6_H_5_OH)) that can potentially serve as both stabilizing and reducing agents in the rapid biosynthesis of Ag/AgCl-NPs-ME through microwave-assisted synthesis. Characterization of the synthesized Ag/AgCl-NPs was performed utilizing a wide range of methods, such as UV–vis, FTIR, XRD, SEM, TEM, and DLS, which collectively confirmed their favorable dispersion and stability. Additionally, these nanoparticles exhibited notable antioxidant activities, demonstrated high inhibitory effects on enzymes associated with diseases, showed strong cytotoxic effects on cancer cells, and were non-toxic to normal human cells. These findings highlight the significant biotechnological potential of Ag/AgCl-NPs-ME.

## Data Availability

The original contributions presented in the study are included in the article. Further inquiries can be directed to the corresponding author.

## References

[1] Xiao J., Wang Z., Liu D., Fu M., Yuan C., Yan T. (2021). Harmful macroalgal blooms (HMBs) in China’s coastal water: Green and golden tides. Harmful Algae.

[2] Oxenford H.A., Cox S.A., van Tussenbroek B.I., Desrochers A. (2021). Challenges of turning the *Sargassum* crisis into gold: Current constraints and implications for the Caribbean. Phycology.

[3] Rosellón-Druker J., Calixto-Pérez E., Escobar-Briones E., González-Cano J., Masiá-Nebot L., Córdova-Tapia F. (2022). A review of a decade of local projects, studies and initiatives of atypical influxes of pelagic *Sargassum* on Mexican Caribbean coasts. Phycology.

[4] Laurens L.M., Lane M., Nelson R.S. (2020). Sustainable seaweed biotechnology solutions for carbon capture, composition, and deconstruction. Trends Biotechnol..

[5] Luna-Pérez Y., Ríos-López L.G., Otero-Tejada E.L., Mejía-Giraldo J.C., Puertas-Mejía M.Á. (2023). *Sargassum filipendula*, a source of bioactive compounds with antioxidant and matrix metalloproteinases inhibition activities in vitro with potential dermocosmetic application. Antioxidants.

[6] Ruangrit K., Chaipoot S., Phongphisutthinant R., Duangjan K., Phinyo K., Jeerapan I., Pekkoh J., Srinuanpan S. (2023). A successful biorefinery approach of macroalgal biomass as a promising sustainable source to produce bioactive nutraceutical and biodiesel. Biomass Conv. Bioref..

[7] Lee M.K., Ryu H., Lee J.Y., Jeong H.H., Baek J., Van J.Y., Kim M.J., Jung W.K., Lee B. (2022). Potential beneficial effects of *Sargassum* spp. in skin aging. Mar. Drugs.

[8] Giantina G., Giriwono P.E., Faridah D.N., Iskandriati D., Andarwulan N. (2020). Water and lipid-soluble component profile of *Sargassum cristaefolium* from different coastal areas in Indonesia with potential for developing functional ingredient. J. Oleo. Sci..

[9] Jing X., Meng X., Wu Z., Ding Y., Peng Y., Shen M., Wang Q. (2022). Sub-acute toxicity of licorice-sargassum extract in Sprague-Dawley rats: Biochemical, histopathological, and pharmacokinetic studies. Chin. Med. J..

[10] Badmus J.A., Oyemomi S.A., Adedosu O.T., Yekeen T.A., Azeez M.A., Adebayo E.A., Lateef A., Badeggi U.M., Botha S., Hussein A.A. (2020). Photo-assisted bio-fabrication of silver nanoparticles using Annona muricata leaf extract: Exploring the antioxidant, anti-diabetic, antimicrobial, and cytotoxic activities. Heliyon.

[11] González-Ballesteros N., Rodríguez-Argüelles M.C., Lastra-Valdor M., González-Mediero G., Rey-Cao S., Grimaldi M., Cavazza A., Bigi F. (2020). Synthesis of silver and gold nanoparticles by *Sargassum muticum* biomolecules and evaluation of their antioxidant activity and antibacterial properties. J. Nanostruct. Chem..

[12] Noman M., Shahid M., Ahmed T., Niazi M.B.K., Hussain S., Song F., Manzoor I. (2020). Use of biogenic copper nanoparticles synthesized from a native *Escherichia* sp. as photocatalysts for azo dye degradation and treatment of textile effluents. Environ. Pollut..

[13] Ezealigo U.S., Ezealigo B.N., Aisida S.O., Ezema F.I. (2021). Iron oxide nanoparticles in biological systems: Antibacterial and toxicology perspective. JCIS Open.

[14] Khan A.U., Malik N., Singh B., Ansari N.H., Rehman M., Yadav A. (2023). Biosynthesis, and characterization of zinc oxide nanoparticles (ZnONPs) obtained from the extract of waste of strawberry. J. Umm Al-Qura Univ. Appl. Sci..

[15] Rathi V.H., Jeice A.R. (2023). Green fabrication of titanium dioxide nanoparticles and their applications in photocatalytic dye degradation and microbial activities. Chem. Phys. Impact..

[16] Ramezani Farani M., Farsadrooh M., Zare I., Gholami A., Akhavan O. (2023). Green synthesis of magnesium oxide nanoparticles and nanocomposites for photocatalytic antimicrobial, antibiofilm and antifungal applications. Catalysts.

[17] Zarenezhad E., Abdulabbas H.T., Marzi M., Ghazy E., Ekrahi M., Pezeshki B., Ghasemian A., Moawad A.A. (2022). Nickel nanoparticles: Applications and antimicrobial role against methicillin-resistant *Staphylococcus aureus* infections. Antibiotics.

[18] Pescuma M., Aparicio F., Zysler R.D., Lima E., Zapata C., Marfetán J.A., Vélez M.L., Ordoñez O.F. (2023). Biogenic selenium nanoparticles with antifungal activity against the wood-rotting fungus *Oligoporus pelliculosus*. Biotechnol. Rep..

[19] Khan M., Mashwani Z.U.R., Ikram M., Raja N.I., Mohamed A.H., Ren G., Omar A.A. (2022). Efficacy of green cerium oxide nanoparticles for potential therapeutic applications: Circumstantial insight on mechanistic aspects. Nanomaterials.

[20] Singh I., Gupta S., Gautam H.K., Dhawan G., Kumar P. (2021). Antimicrobial, radical scavenging, and dye degradation potential of nontoxic biogenic silver nanoparticles using *Cassia fistula* pods. Chem. Pap..

[21] Quintero-Quiroz C., Acevedo N., Zapata-Giraldo J., Botero L.E., Quintero J., Zárate-Triviño D., Saldarriaga J., Pérez V.Z. (2019). Optimization of silver nanoparticle synthesis by chemical reduction and evaluation of its antimicrobial and toxic activity. Biomater. Res..

[22] Jara N., Milán N.S., Rahman A., Mouheb L., Boffito D.C., Jeffryes C., Dahoumane S.A. (2021). Photochemical synthesis of gold and silver nanoparticles—A review. Molecules.

[23] Nasretdinova G.R., Fazleeva R.R., Mukhitova R.K., Nizameev I.R., Kadirov M.K., Ziganshina A.Y., Yanilkin V.V. (2015). Electrochemical synthesis of silver nanoparticles in solution. Electrochem. Commun..

[24] Das M., Patowary K., Vidya R., Malipeddi H. (2016). Microemulsion synthesis of silver nanoparticles using biosurfactant extracted from *Pseudomonas aeruginosa* MKVIT3 strain and comparison of their antimicrobial and cytotoxic activities. IET Nanobiotechnol..

[25] Joseph S., Mathew B. (2015). Microwave-assisted green synthesis of silver nanoparticles and the study on catalytic activity in the degradation of dyes. J. Mol. Liq..

[26] Adedokun O., Ntungwe E.N., Viegas C., Adesina Ayinde B., Barboni L., Maggi F., Saraiva L., Rijo P., Fonte P. (2022). Enhanced anticancer activity of *Hymenocardia acida* stem bark extract loaded into PLGA nanoparticles. Pharmaceuticals.

[27] Revathi N., Sankarganesh M., Raja J.D., Rajakanna J., Senthilkumar O. (2023). Green synthesis of *Plectranthus amboinicus* leaf extract incorporated fine-tuned manganese dioxide nanoparticles: Antimicrobial and antioxidant activity. Inorg. Chem. Commun..

[28] Acar C.A., Pehlivanoglu S., Yesilot S., Uzuner S.Y. (2023). Microwave-assisted biofabrication of silver nanoparticles using *Helichrysum arenarium* flower extract: Characterization and biomedical applications. Biomass Conv. Bioref..

[29] Balaraman P., Balasubramanian B., Kaliannan D., Durai M., Kamyab H., Park S., Chelliapan S., Lee C.T., Maluventhen V., Maruthupandian A. (2020). Phyco-synthesis of silver nanoparticles mediated from marine algae *Sargassum myriocystum* and its potential biological and environmental applications. Waste Biomass Valor..

[30] Thiurunavukkarau R., Shanmugam S., Subramanian K., Pandi P., Muralitharan G., Arokiarajan M., Kasinathan K., Sivaraj A., Kalyanasundaram R., AlOmar S.Y. (2022). Silver nanoparticles synthesized from the seaweed *Sargassum polycystum* and screening for their biological potential. Sci. Rep..

[31] Deepak P., Amutha V., Birundha R., Sowmiya R., Kamaraj C., Balasubramanian V., Balasubramani G., Aiswarya D., Arul D., Perumal P. (2018). Facile green synthesis of nanoparticles from brown seaweed *Sargassum wightii* and its biological application potential. Adv. Nat. Sci. Nanosci. Nanotechnol..

[32] Mmola M., Roes-Hill M.L., Durrell K., Bolton J.J., Sibuyi N., Meyer M.E., Beukes D.R., Antunes E. (2016). Enhanced antimicrobial and anticancer activity of silver and gold nanoparticles synthesised using *Sargassum incisifolium* aqueous extracts. Molecules.

[33] Pekkoh J., Phinyo K., Thurakit T., Lomakool S., Duangjan K., Ruangrit K., Pumas C., Jiranusornkul S., Yooin W., Cheirsilp B. (2022). Lipid profile, antioxidant and antihypertensive activity, and computational molecular docking of diatom fatty acids as ACE inhibitors. Antioxidants.

[34] Pekkoh J., Ruangrit K., Pumas C., Duangjan K., Chaipoot S., Phongphisutthinant R., Jeerapan I., Sawangrat K., Pathom-aree W., Srinuanpan S. (2023). Transforming microalgal *Chlorella* biomass into cosmetically and nutraceutically protein hydrolysates using high-efficiency enzymatic hydrolysis approach. Biomass Conv. Bioref..

[35] Phinyo K., Ruangrit K., Pekkoh J., Tragoolpua Y., Kaewkod T., Duangjan K., Pumas C., Suwannarach N., Kumla J., Pathom-Aree W. (2022). Naturally occurring functional ingredient from filamentous thermophilic cyanobacterium *Leptolyngbya* sp. KC45: Phytochemical characterizations and their multiple bioactivities. Antioxidants.

[36] Sharifi-Rad M., Pohl P. (2020). Synthesis of biogenic silver nanoparticles (Agcl-NPs) using a *Pulicaria vulgaris* gaertn. aerial part extract and their application as antibacterial, antifungal and antioxidant agents. Nanomaterials.

[37] Hassan K.T., Ibraheem I.J., Hassan O.M., Obaid A.S., Ali H.H., Salih T.A., Kadhim M.S. (2021). Facile green synthesis of Ag/AgCl nanoparticles derived from *Chara* algae extract and evaluating their antibacterial activity and synergistic effect with antibiotics. J. Environ. Chem. Eng..

[38] Shah M., Nawaz S., Jan H., Uddin N., Ali A., Anjum S., Giglioli-Guivarc’h N., Hano C., Abbasi B.H. (2020). Synthesis of bio-mediated silver nanoparticles from *Silybum marianum* and their biological and clinical activities. Mater. Sci. Eng. C..

[39] Rajkumar R., Ezhumalai G., Gnanadesigan M. (2021). A green approach for the synthesis of silver nanoparticles by *Chlorella vulgaris* and its application in photocatalytic dye degradation activity. Environ. Technol. Innov..

[40] Moshahary S., Mishra P. (2021). Synthesis of silver nanoparticles (AgNPs) using culinary banana peel extract for the detection of melamine in milk. J. Food Sci. Technol..

[41] Bakht Dalir S.J., Djahaniani H., Nabati F., Hekmati M. (2020). Characterization and the evaluation of antimicrobial activities of silver nanoparticles biosynthesized from *Carya illinoinensis* leaf extract. Heliyon.

[42] Xu L., Wang Y.Y., Huang J., Chen C.Y., Wang Z.X., Xie H. (2020). Silver nanoparticles: Synthesis, medical applications and biosafety. Theranostics.

[43] Farshad M., Rasaiah J. (2023). Kinetics of nanoparticle nucleation, growth, coalescence and aggregation: A theoretical study of (Ag) n nanoparticle formation based on population balance modulated by ligand binding. Chem. Phys..

[44] Vasquez R.D., Apostol J.G., de Leon J.D., Mariano J.D., Mirhan C.M.C., Pangan S.S., Reyes A.G.M., Zamora E.T. (2016). Polysaccharide-mediated green synthesis of silver nanoparticles from *Sargassum siliquosum* JG Agardh: Assessment of toxicity and hepatoprotective activity. OpenNano.

[45] Rao Y., Wen Q., Liu R., He M., Jiang Z., Qian K., Zhou C., Li J., Du H., Ouyang H. (2020). PL-S2, a homogeneous polysaccharide from Radix *Puerariae lobatae*, attenuates hyperlipidemia via farnesoid X receptor (FXR) pathway-modulated bile acid metabolism. Int. J. Biol. Macromol..

[46] Pekkoh J., Lomakool S., Chankham J., Duangjan K., Thurakit T., Phinyo K., Ruangrit K., Tragoolpua Y., Pumas C., Pathom-aree W. (2022). Maximizing biomass productivity of cyanobacterium *Nostoc* sp. through high-throughput bioprocess optimization and application in multiproduct biorefinery towards a holistic zero waste. Biomass Conv. Bioref..

[47] Kashyap M., Samadhiya K., Ghosh A., Anand V., Lee H., Sawamoto N., Ogura A., Ohshita Y., Shirage P.M., Bala K. (2021). Synthesis, characterization and application of intracellular Ag/AgCl nanohybrids biosynthesized in *Scenedesmus* sp. as neutral lipid inducer and antibacterial agent. Environ. Res..

[48] Kiran M.S., Betageri V.S., Kumar C.R., Vinay S.P., Latha M.S. (2020). In-vitro antibacterial, antioxidant and cytotoxic potential of silver nanoparticles synthesized using novel *Eucalyptus tereticornis* leaves extract. J. Inorg. Organomet. Polym..

[49] Vijayakumar S., Divya M., Vaseeharan B., Chen J., Biruntha M., Silva L.P., Duran-Lara E.F., Shreema K., Ranjan S., Dasgupta N. (2021). Biological compound capping of silver nanoparticle with the seed extracts of blackcumin (*Nigella sativa*): A potential antibacterial, antidiabetic, anti-inflammatory, and antioxidant. J. Inorg. Organomet. Polym..

[50] Ajayi E., Afolayan A. (2017). Green synthesis, characterization and biological activities of silver nanoparticles from alkalinized *Cymbopogon citratus* Stapf. Adv. Nat. Sci. Nanosci. Nanotechnol..

[51] Mahendran G., Kumari B.R. (2016). Biological activities of silver nanoparticles from *Nothapodytes nimmoniana* (Graham) Mabb. fruit extracts. Food Sci. Hum. Wellness.

[52] Elemike E.E., Fayemi O.E., Ekennia A.C., Onwudiwe D.C., Ebenso E.E. (2017). Silver nanoparticles mediated by *Costus afer* leaf extract: Synthesis, antibacterial, antioxidant and electrochemical properties. Molecules.

[53] Otunola G.A., Afolayan A.J. (2018). In vitro antibacterial, antioxidant and toxicity profile of silver nanoparticles green-synthesized and characterized from aqueous extract of a spice blend formulation. Biotechnol. Biotechnol. Equip..

[54] Tanase C., Berta L., Mare A., Man A., Talmaciu A.I., Roșca I., Mircia E., Volf I., Popa V.I. (2020). Biosynthesis of silver nanoparticles using aqueous bark extract of *Picea abies* L. and their antibacterial activity. Eur. J. Wood Wood Prod..

[55] Chokshi K., Pancha I., Ghosh T., Paliwal C., Maurya R., Ghosh A., Mishra S. (2016). Green synthesis, characterization and antioxidant potential of silver nanoparticles biosynthesized from de-oiled biomass of thermotolerant oleaginous microalgae *Acutodesmus dimorphus*. RSC Adv..

[56] Rajurkar N.S., Hande S.M. (2011). Estimation of phytochemical content and antioxidant activity of some selected traditional Indian medicinal plants. Indian J. Pharm. Sci..

[57] Moteriya P., Padalia H., Chanda S. (2017). Characterization, synergistic antibacterial and free radical scavenging efficacy of silver nanoparticles synthesized using *Cassia roxburghii* leaf extract. J. Genet. Eng. Biotechnol..

[58] Samari F., Parkhari P., Eftekhar E., Mohseni F., Yousefinejad S. (2019). Antioxidant, cytotoxic and catalytic degradation efficiency of controllable phyto-synthesised silver nanoparticles with high stability using *Cordia myxa* extract. J. Exp. Nanosci..

[59] Suwan T., Wanachantararak P., Khongkhunthian S., Okonogi S. (2018). Antioxidant activity and potential of *Caesalpinia sappan* aqueous extract on synthesis of silver nanoparticles. Drug Discov. Ther..

[60] Jesumani V., Du H., Pei P., Aslam M., Huang N. (2020). Comparative study on skin protection activity of polyphenol-rich extract and polysaccharide-rich extract from *Sargassum vachellianum*. PLoS ONE.

[61] Basavegowda N., Idhayadhulla A., Lee Y.R. (2014). Tyrosinase inhibitory activity of silver nanoparticles treated with *Hovenia dulcis* fruit extract: An in vitro study. Mater. Lett..

[62] Abbas Q., Saleem M., Phull A.R., Rafiq M., Hassan M., Lee K.H., Seo S.Y. (2017). Green synthesis of silver nanoparticles using *Bidens frondosa* extract and their tyrosinase activity. Iran. J. Pharm. Res..

[63] Ceylan R., Demirbas A., Ocsoy I., Aktumsek A. (2021). Green synthesis of silver nanoparticles using aqueous extracts of three *Sideritis* species from Turkey and evaluations bioactivity potentials. Sustain. Chem. Pharm..

[64] Talapko J., Matijević T., Juzbašić M., Antolović-Požgain A., Škrlec I. (2020). Antibacterial activity of silver and its application in dentistry, cardiology and dermatology. Microorganisms.

[65] Gonzalez C., Rosas-Hernandez H., Ramirez-Lee M.A., Salazar-García S., Ali S.F. (2016). Role of silver nanoparticles (AgNPs) on the cardiovascular system. Arch. Toxicol..

[66] Ramirez-Lee M.A., Aguirre-Bañuelos P., Martinez-Cuevas P.P., Espinosa-Tanguma R., Chi-Ahumada E., Martinez-Castañon G.A., Gonzalez C. (2018). Evaluation of cardiovascular responses to silver nanoparticles (AgNPs) in spontaneously hypertensive rats. Nanomed. Nanotechnol. Biol. Med..

[67] Cao G., Lin H., Kannan P., Wang C., Zhong Y., Huang Y., Guo Z. (2018). Enhanced antibacterial and food simulant activities of silver nanoparticles/polypropylene nanocomposite films. Langmuir.

[68] Gomes H.I., Martins C.S., Prior J.A. (2021). Silver nanoparticles as carriers of anticancer drugs for efficient target treatment of cancer cells. Nanomaterials.

[69] Peña-Morán O.A., Villarreal M.L., Álvarez-Berber L., Meneses-Acosta A., Rodríguez-López V. (2016). Cytotoxicity, post-treatment recovery, and selectivity analysis of naturally occurring podophyllotoxins from *Bursera fagaroides* var. fagaroides on breast cancer cell lines. Molecules.

[70] Weerapreeyakul N., Nonpunya A., Barusrux S., Thitimetharoch T., Sripanidkulchai B. (2012). Evaluation of the anticancer potential of six herbs against a Hepatoma cell line. Chin. Med..

[71] Rashidi M., Seghatoleslam A., Namavari M., Amiri A., Fahmidehkar M.A., Ramezani A., Eftekhar E., Hosseini A., Erfani N., Fakher S. (2017). Selective cytotoxicity and apoptosis-induction of *Cyrtopodion scabrum* extract against digestive cancer cell lines. Int. J. Cancer Manag..

[72] Krzywik J., Mozga W., Aminpour M., Janczak J., Maj E., Wietrzyk J., Tuszyński J.A., Huczyński A. (2020). Synthesis, antiproliferative activity and molecular docking studies of novel doubly modified colchicine amides and sulfonamides as anticancer agents. Molecules.

[73] Verma J., Warsame C., Seenivasagam R.K., Katiyar N.K., Aleem E., Goel S. (2023). Nanoparticle-mediated cancer cell therapy: Basic science to clinical applications. Cancer Metastasis Rev..

[74] Yuan Y.G., Zhang S., Hwang J.Y., Kong I.K. (2018). Silver nanoparticles potentiates cytotoxicity and apoptotic potential of camptothecin in human cervical cancer cells. Oxid. Med. Cell. Longev..

[75] Kovács D., Igaz N., Gopisetty M.K., Kiricsi M. (2022). Cancer therapy by silver nanoparticles: Fiction or reality?. Int. J. Mol. Sci..

[76] Holmila R.J., Vance S.A., King S.B., Tsang A.W., Singh R., Furdui C.M. (2019). Silver nanoparticles induce mitochondrial protein oxidation in lung cells impacting cell cycle and proliferation. Antioxidants.

[77] Takáč P., Michalková R., Čižmáriková M., Bedlovičová Z., Balážová Ľ., Takáčová G. (2023). The Role of silver nanoparticles in the diagnosis and treatment of cancer: Are there any perspectives for the future?. Life.

[78] Pekkoh J., Duangjan K., Phinyo K., Kaewkod T., Ruangrit K., Thurakit T., Pumas C., Pathom-aree W., Cheirsilp B., Gu W. (2023). Turning waste CO_2_ into value-added biorefinery co-products using cyanobacterium *Leptolyngbya* sp. KC45 as a highly efficient living photocatalyst. Chem. Eng. J..

